# 7-Substituted 2-Nitro-5,6-dihydroimidazo[2,1-*b*][1,3]oxazines: Novel Antitubercular Agents Lead to a New Preclinical Candidate for Visceral Leishmaniasis

**DOI:** 10.1021/acs.jmedchem.7b00034

**Published:** 2017-05-01

**Authors:** Andrew M. Thompson, Patrick D. O’Connor, Andrew J. Marshall, Vanessa Yardley, Louis Maes, Suman Gupta, Delphine Launay, Stephanie Braillard, Eric Chatelain, Scott G. Franzblau, Baojie Wan, Yuehong Wang, Zhenkun Ma, Christopher B. Cooper, William A. Denny

**Affiliations:** † Auckland Cancer Society Research Centre, School of Medical Sciences, The University of Auckland, Private Bag 92019, Auckland 1142, New Zealand; ‡ Faculty of Infectious & Tropical Diseases, London School of Hygiene & Tropical Medicine, Keppel Street, London WC1E 7HT, United Kingdom; § Laboratory for Microbiology, Parasitology and Hygiene, Faculty of Pharmaceutical, Biomedical and Veterinary Sciences, University of Antwerp, Universiteitsplein 1, B-2610 Antwerp, Belgium; ∥ Division of Parasitology, CSIR—Central Drug Research Institute, Lucknow 226031, India; ⊥ Drugs for Neglected Diseases *initiative*, 15 Chemin Louis Dunant, 1202 Geneva, Switzerland; # Institute for Tuberculosis Research, College of Pharmacy, University of Illinois at Chicago, 833 South Wood Street, Chicago, Illinois 60612, United States; ∇ Global Alliance for TB Drug Development, 40 Wall Street, New York 10005, United States

## Abstract

Within a backup program for the clinical investigational agent pretomanid (PA-824), scaffold hopping from delamanid inspired the discovery of a novel class of potent antitubercular agents that unexpectedly possessed notable utility against the kinetoplastid disease visceral leishmaniasis (VL). Following the identification of delamanid analogue DNDI-VL-2098 as a VL preclinical candidate, this structurally related 7-substituted 2-nitro-5,6-dihydroimidazo[2,1-*b*][1,3]oxazine class was further explored, seeking efficacious backup compounds with improved solubility and safety. Commencing with a biphenyl lead, bioisosteres formed by replacing one phenyl by pyridine or pyrimidine showed improved solubility and potency, whereas more hydrophilic side chains reduced VL activity. In a *Leishmania donovani* mouse model, two racemic phenylpyridines (**71** and **93**) were superior, with the former providing >99% inhibition at 12.5 mg/kg (b.i.d., orally) in the *Leishmania infantum* hamster model. Overall, the 7*R* enantiomer of **71** (**79**) displayed more optimal efficacy, pharmacokinetics, and safety, leading to its selection as the preferred development candidate.

**Figure:**
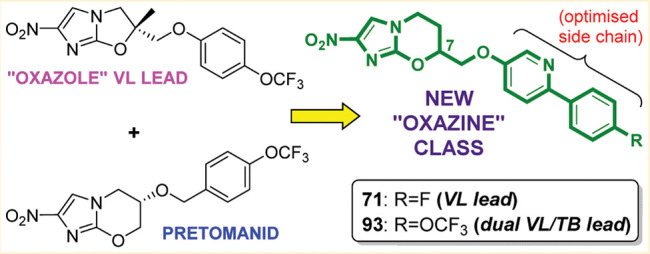

## ■ INTRODUCTION

**Figure 1:**
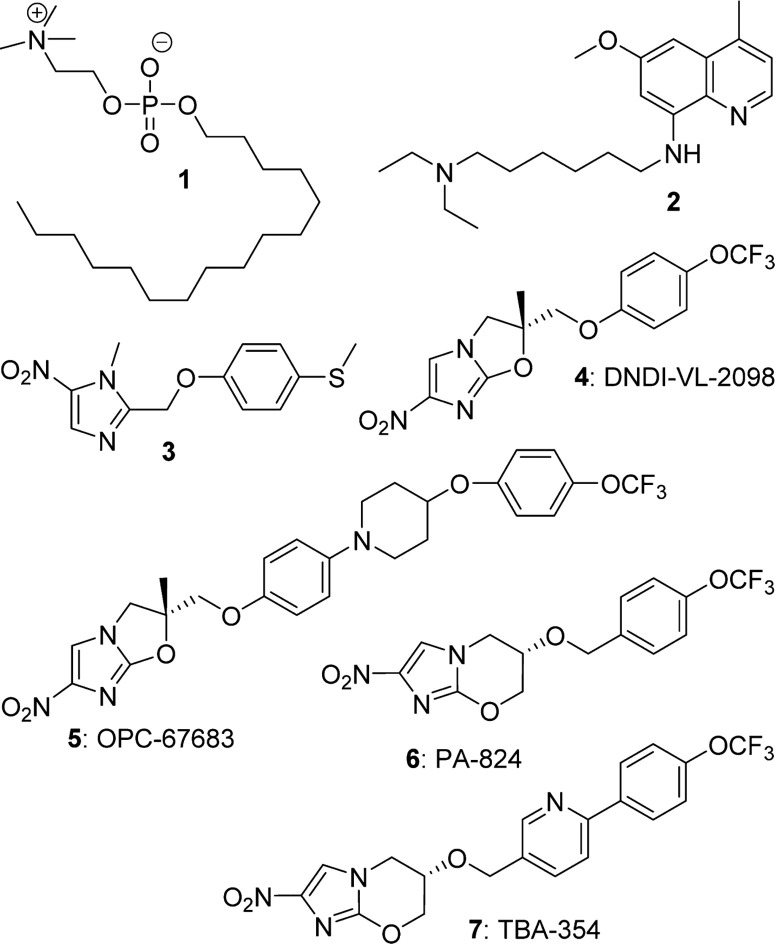
Structures of antitubercular or antileishmanial agents.

The neglected tropical disease visceral leishmaniasis (VL) is the second deadliest parasitic disorder (after malaria), being most prevalent in Brazil, Sudan, Ethiopia, and the Indian subcontinent, with an estimated 350 million people at risk of infection.^1^ Transmitted by sand flies, the disease first manifests as an irregular fever, anemia, leukopenia, and hepatosplenomegaly and is usually fatal within two years if left untreated.^2^ About 300000 new cases arise annually, almost half in children, and at least 35 countries have reported the occurrence of HIV coinfection (with up to 34% incidence), which gives a significantly higher mortality rate.^3,4^ Unfortunately, none of the existing VL drugs (antimonials, paromomycin, liposomal amphotericin B, or miltefosine **1**; see Figure 1) is universally effective nor free from further drawbacks such as parenteral administration (for all except **1**), toxicity, high cost, and emerging resistance.^5^ Furthermore, there is no available vaccine despite renewed efforts.^6^ Clinical investigation of the orally active aminoquinoline sitamaquine (**2**) has been abandoned due to its toxicity and less satisfactory efficacy,^7^ and new phase II trials of the repositioned oral agent fexinidazole (**3**)^8^ for VL have also been interrupted due to patient relapses.^9^ With no other candidates under clinical evaluation at present, there is a desperate need for the development of more effective, safe, and affordable oral remedies for VL.

**Figure 2:**
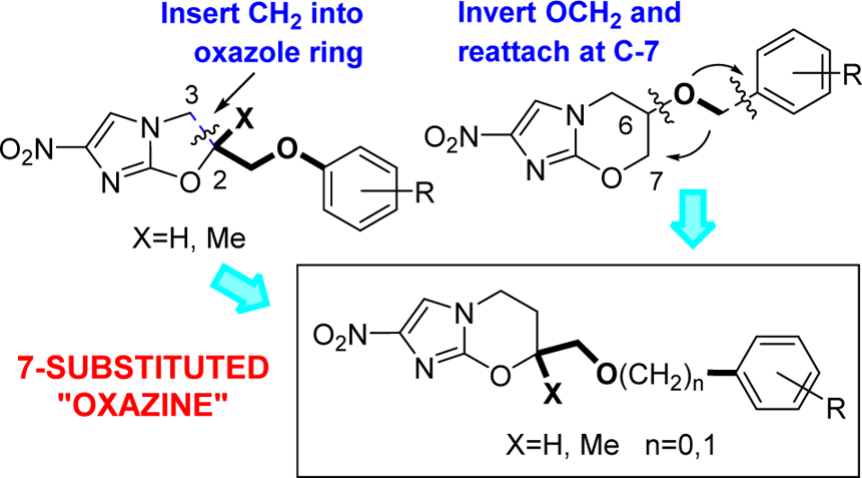
Scaffold hopping to 7-substituted 2-nitroimidazooxazines.

We have recently reported that phenotypic screening by the Drugs for Neglected Diseases *initiative* (DND*i*) of some nitroimidazole derivatives arising from our early studies with the TB Alliance unexpectedly led to the identification of DNDI-VL-2098 (**4**) as a preclinical candidate for VL.^10,11^ Our opening assignment with TB Alliance had been to prepare and evaluate novel nitroheterobicyclic analogues of the tuberculosis (TB) drugs delamanid (**5**) and pretomanid (PA-824, **6**),^12,13^ seeking a possible third active scaffold for the construction of a backup series. However, among the fused 5/6 ring systems examined, only the metabolically labile 2-nitroimidazothiazines retained significant antitubercular potency,^14^ returning our attention to the original oxazine class where we uncovered heterobiaryl derivatives of **6** with better efficacy (e.g., TBA-354, **7**).^15,16^ One important consideration in the design of a superior second-generation TB candidate was the potential for cleavage of the aromatic side chain via oxidative metabolism of the 6-oxymethylene linker; therefore, several alternative linker and steric protection strategies were explored, albeit with limited success.^17–19^ A final, more innovative way to address this issue was to invoke a scaffold hopping approach^20^ by relocating aromatic side chains from the 6-position to the 7-position of the 2-nitroimidazooxazine core, with attachment via the same inverted linker (CH_2_OR) that was present in 6-nitroimidazooxazole **5**. This was equivalent to a one carbon expansion of the oxazole ring between C-2 and C-3 (Figure 2). The rationale for this design concept stemmed from initial evidence^21^ that delamanid (**5**) was highly stable toward metabolism as well as from a report^22^ that 7-methyl derivatives of **6** retained excellent antitubercular potency, suggesting that such an approach merited investigation.

Serendipitously, we soon discovered^23^ that this novel “7-substituted oxazine” class not only showed considerable promise for TB (as later confirmed by others^24,25^), it also displayed potent antileishmanial activity comparable to the 6-nitroimidazooxazoles in early screening assays. Therefore, following the success with **4**, this new series was similarly repositioned for VL as part of an extensive backup program run in collaboration with DND*i*. In this paper, we first highlight some critical VL hit to lead assessments on the original subset of compounds that had been prepared for TB. We then detail the findings of our lead optimization study directed at developing backups to **4** having an improved physicochemical/pharmacological profile and better safety, which culminated in the selection of a new preclinical candidate for VL. Finally, in light of these encouraging results and the excellent activities of this novel 7-substituted 2-nitroimidazooxazine class against both TB and Chagas disease, we point to related analogues that might be worthy of further assessment for the latter applications.

## ■ CHEMISTRY

**Scheme 1a:**
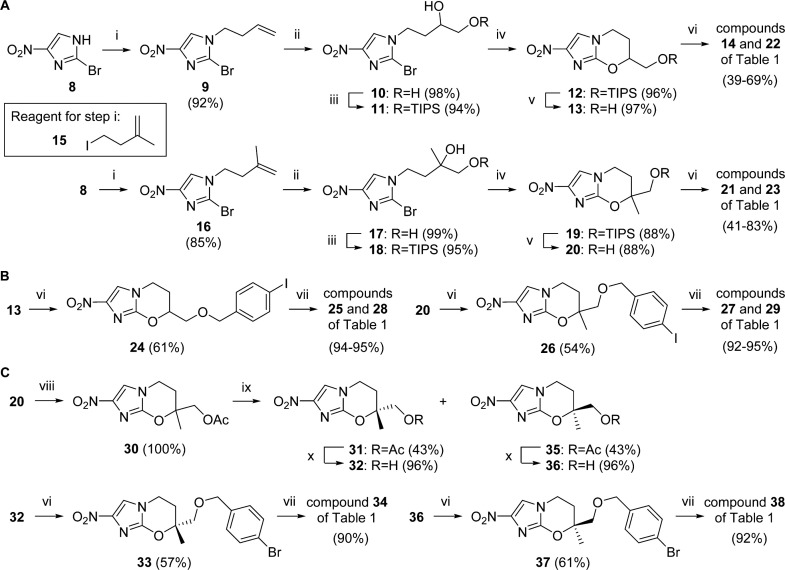
^a^Reagents and conditions: (i) Br(CH_2_)_2_CH=CH_2_ or **15**, K_2_CO_3_, DMF, 60–73 °C, 4.5–11 h; (ii) OsO_4_, NMO, CH_2_Cl_2_,20 °C, 4 h; (iii) TIPSCl, imidazole, DMF, 20 °C, 2–3d;(iv)NaH, DMF, 0–20 °C, 3.4 h (for **12**), or 0–20 °C, 2.5 h then 46 °C,3h(for **19**); (v) 1% HCl in 95% EtOH, 20 or 44 °C, 1.5–3d;(vi)ArCH_2_Br or 4-BnOBnCl, NaH, DMF, 0–20 °C, 2.5–7 h; (vii) ArB(OH)_2_, toluene, EtOH, 2 M Na_2_CO_3_, Pd(dppf)Cl_2_ under N_2_, 86–90 °C, 20–75 min; (viii) Ac_2_O, pyridine, 20 °C, 38 h; (ix) preparative chiral HPLC (see text); (x) K_2_CO_3_, aq MeOH, 20 °C, 4 h.

To rapidly access some initial examples, the racemic 7-H and 7-methyl alcohol intermediates, **13** and **20**, were first sought (Scheme 1A). These could be obtained in very good overall yield (62–79%) via similar five-step reaction sequences, starting with base catalyzed alkylation of 2-bromo-4-nitroimidazole (**8**) using 4-bromobut-1-ene or 4-iodo-2-methylbut-1-ene^26^ (**15**). Dihydroxylation of the resulting alkene (OsO_4_/NMO), selective TIPS protection of the primary hydroxyl group, sodium hydride-induced ring closure, and acid-catalyzed desilylation^27^ completed the synthesis of both alcohols, although in the case of **20**, the final two steps required gentle warming. The benzyl ether targets **14**, **21–23**, **25**, and **27–29** were then formed by standard alkylation and Suzuki coupling methodology (Scheme 1A,B). Next, the two enantiomers of early TB lead **29** (**34** and **38**) were also generated via preparative chiral HPLC separation of the 7*R* and 7*S* forms of acetate derivative **30**, followed by hydrolysis to the chiral alcohols (**32** and **36**) and elaboration as before (Scheme 1C). Here, the absolute configurations of **34** and **38** were subsequently established through an independent chiral synthesis of the 7*R* enantiomer (see the Supporting Information), involving alkylation of **8** with the iodide derived from 2-[(2*R*)-2-methyl-1,4-dioxaspiro[4.5]decan-2-yl]ethan-1-ol.^28^

**Scheme 2a:**
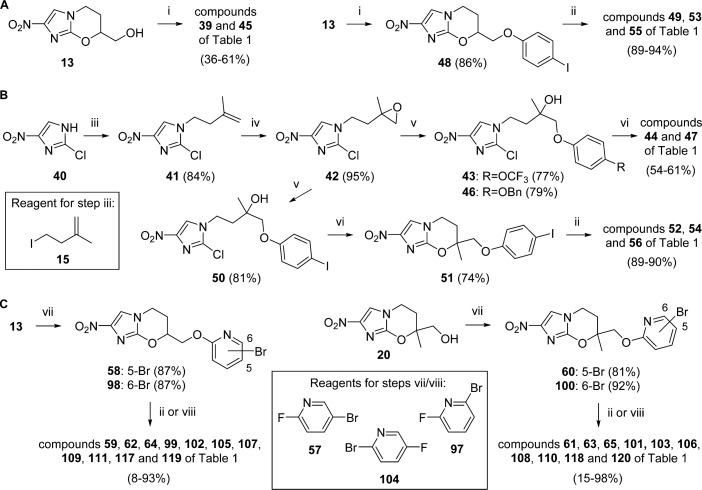
^a^Reagents and conditions: (i) ArOH, DEAD, PPh_3_, THF, 0–20 °C, 15–51 h; (ii) ArB(OH)_2_, DMF, (toluene, EtOH), 2 M Na_2_CO_3_ or 2 M KHCO_3_, Pd(dppf)Cl_2_ under N_2_, 70–91 °C, 1.5–5 h; (iii) **15**, K_2_CO_3_, DMF, 73 °C, 14 h; (iv) *m*-CPBA, Na_2_HPO_4_, CH_2_Cl_2_, 0–20 °C, 18 h; (v) ArOH, K_2_CO_3_, MEK, 82–83 °C, 8–10 h; (vi) NaH, DMF, 0–20 °C, 2–2.5 h; (vii) **57** or **97**, NaH, DMF, 0–20 °C, 2.3–3 h; (viii) bis(pinacolato)diboron, KOAc, DMF, Pd(dppf)Cl_2_ under N_2_,84–90 °C, 3.5 h, then **104**,2MNa_2_CO_3_, Pd(dppf)Cl_2_ under N_2_,85–90 °C, 2.5–3.5 h.

Mitsunobu coupling of alcohol **13** with appropriate phenols (Scheme 2A) successfully led to the 7-H phenyl ethers **39** and **45**, together with the 4-iodo analogue **48**; the latter enabled biphenyl derivatives **49** and **53**, following Suzuki couplings. However, because Mitsunobu reactions were expected to be more problematic for the sterically hindered 7-methyl alcohol **20**,^29^ a different approach was employed to prepare compounds **44** and **47** (Scheme 2B). Commencing with 2-chloro-4-nitroimidazole (**40**), alkylation with iodide **15**^26^ and buffered reaction of alkene **41** with *m*-CPBA provided epoxide **42** in high yield (80%). Ring opening of **42** with phenols (K_2_CO_3_, MEK, 82 °C) then gave alcohol intermediates that could be ring closed to 7-substituted oxazines, as above. An attempt to combine the last two steps in one pot^30^ (by exposing **42** to 1.2 equiv of NaH and 4-(trifluoromethoxy)phenol in DMF at 75–86 °C) led to markedly inferior results (27% **44**, with 30% **43**); equally, ring opening of **42** with 4-iodophenol in DMF (K_2_CO_3_,83 °C, 8 h) also gave a lower yield of **50** (60%) due to partial displacement of the 2-chlorine. Suzuki couplings on the ring-closed iodide **51** readily furnished biphenyl derivatives **52** and **54**; terminal fluoropyridines **55** and **56** were similarly obtained from **48** and **51** through the use of a weaker base (KHCO_3_).

The assembly of various biaryl side chains featuring a proximal 2-pyridine ring was typically quite straightforward (Scheme 2C). Bromo-2-pyridinyl ethers (**58**, **60**, **98**, and **100**) were easily formed^17^ via sodium hydride-catalyzed S_N_Ar reactions of alcohols **13** and **20** with the fluoropyridines **57** and **97**, and Suzuki couplings then supplied the phenylpyridine or bipyridine targets in generally high yields (62–98%). Nevertheless, it proved very challenging to prepare analogues **105** and **106** having a 2-pyridine terminal ring. One-pot treatment of bromides **58** and **60** with bis(pinacolato)diboron (to generate the boronate ester), followed by in situ Suzuki coupling^31^ with 2-bromo-5-fluoropyridine, gave **105** and **106** in poor yields (8–15%). However, a copper(I)-facilitated Suzuki approach,^32^ designed to mitigate facile protodeboronation of the required 2-pyridyl boronate, was not any better (15% yield of **106**).

**Scheme 3a:**
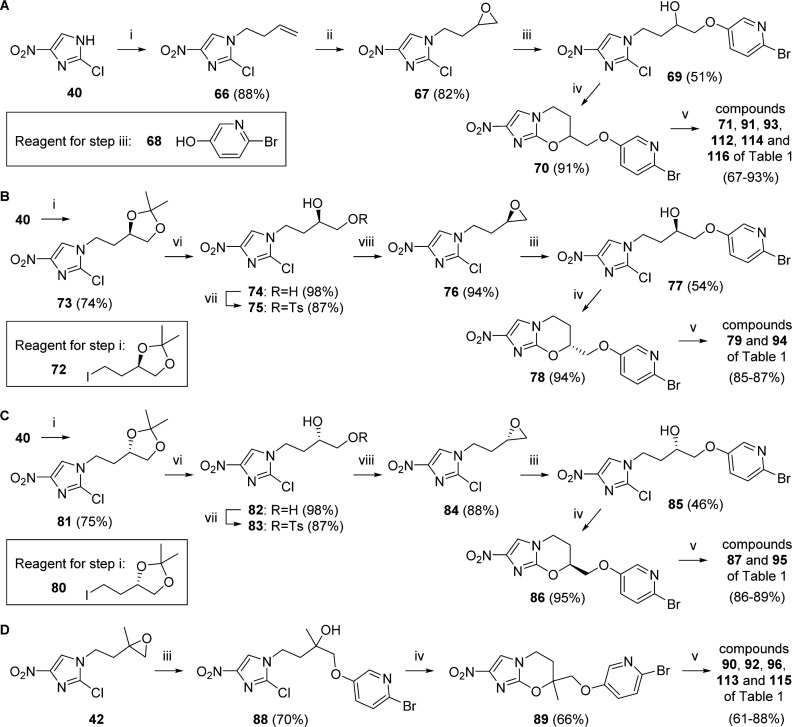
^a^Reagents and conditions: (i) Br(CH_2_)_2_CH=CH_2_ or **72** or **80**, K_2_CO_3_, DMF, 70–74 °C, 19–72 h; (ii) *m*-CPBA, CH_2_Cl_2_,0–20 °C, 34 h; (iii) **68**, K_2_CO_3_, MEK, 81–84 °C, 19–42 h; (iv) NaH, DMF, 0–20 °C, 2.5–3.5 h; (v) ArB(OH)_2_, DMF, (toluene, EtOH), 2 M Na_2_CO_3_ or 2 M KHCO_3_, Pd(dppf)Cl_2_ under N_2_,70–90 °C, 2–4h; (vi) 1 MHCl, MeOH, 0–20 °C, 6 h; (vii) TsCl, pyridine, –10 to 20 °C, 13–15 h; (viii) DBU, CH_2_Cl_2_, 0–20 °C, 8–9 h.

For more efficient synthesis of 7-H biaryl analogues having a proximal 3-pyridine ring, an epoxide-opening strategy (Scheme 3A) was preferred over the Mitsunobu route described above. Epoxide **67** was obtained in 72% optimized yield from 2-chloro-4-nitroimidazole (**40**), via alkene **66**; in this case, the slow epoxidation step was best achieved under nonbuffered conditions at higher concentration (with initial cooling). Ring opening of **67** with 6-bromopyridin-3-ol (**68**) (K_2_CO_3_, MEK, 81–82 °C) gave mainly alcohol **69** (51% using 2 equiv for 35 h, or 57% from 4 equiv and 14 h), together with small amounts of the oxazine **70** (6–12%). Ring closure of purified **69** (NaH, DMF, 0–20 °C) then gave additional **70** in excellent yield (91%). Comparable results were obtained for scale-up of **39** from **67** (62%) as well as for reaction of epoxide **42** with pyridinol **68** and ring closure, leading to oxazine **89** (Scheme 3D). As expected, bromides **70** and **89** both proved to be excellent substrates for Suzuki couplings to access the remaining racemic phenylpyridine and bipyridine targets.

By alkylating 2-chloro-4-nitroimidazole (**40**) with (4*R*)-4-(2-iodoethyl)-2,2-dimethyl-1,3-dioxolane^33^ (**72**) (or its optical isomer, **80**^33^), it was possible to transform the above racemic route into a viable chiral synthesis for delivery of both enantiomers of two advanced leads, **71** and **93** (Scheme 3B,C). The two chiral acetal products **73** and **81** were readily converted into the *R* and *S* enantiomers of epoxide **67** (**76** and **84**) by successive hydrolysis (to diols **74** and **82**), tosylation at the primary hydroxyl, and internal substitution to form the oxirane ring (DBU). These chiral epoxides were then elaborated to the final products by reaction with **68**, ring closure, and Suzuki coupling, as previously described.

**Scheme 4a:**
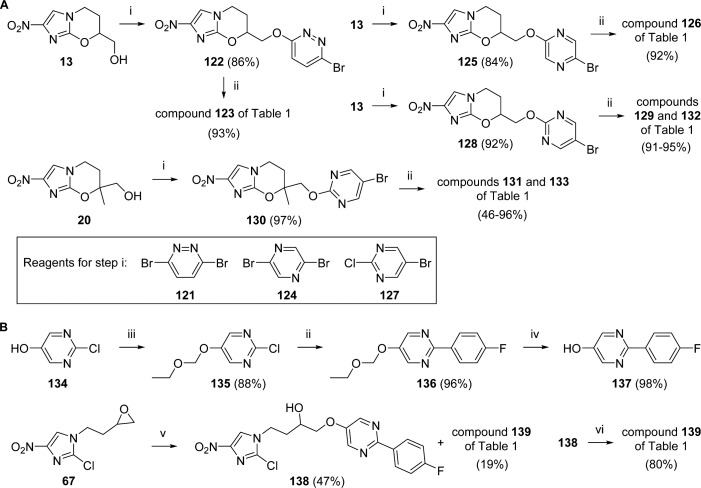
^a^Reagents and conditions: (i) **121** or **124** or **127**, NaH, DMF, 0–20 °C, 2.5–3 h; (ii) ArB(OH)_2_, DMF, toluene, EtOH, 2 M Na_2_CO_3_ or 2 M KHCO_3_, Pd(dppf)Cl_2_ under N_2_,80–90 °C, 2.3–3.5 h; (iii) EtOCH_2_Cl, K_2_CO_3_, DMF, 20 °C, 16 h; (iv) 1.25 M HCl in MeOH, 53 °C, 4 h; (v) **137**, K_2_CO_3_, MEK, 83 °C, 24 h; (vi) NaH, DMF, 0–20 °C, 3 h.

The preparation of biaryl congeners **123**, **126**, **129**, **131–133**, and **139** in which the first ring was pyridazine, pyrazine, or pyrimidine followed similar procedures to those developed for the pyridine analogues. Thus, sodium hydride-induced S_N_Ar reactions of alcohols **13** and **20** with haloheterocycles **121**, **124**, and **127** readily provided the bromoheteroaryl ether intermediates needed for final step Suzuki couplings (Scheme 4A). However, the remaining arylpyrimidine target (**139**) required prior assembly of the biaryl side chain. Initial protection of 2-chloropyrimidin-5-ol (**134**) as an ethoxymethyl ether derivative (**135**), followed by Suzuki coupling and acidic deprotection, supplied arylpyrimidinol **137** in excellent yield (83% from **134**; Scheme 4B). Reaction of **137** with epoxide **67** produced a 5:2 mixture of the alcohol **138** and the ring closed oxazine (**139**); treatment of **138** with sodium hydride then completed this synthesis.

**Scheme 5a:**
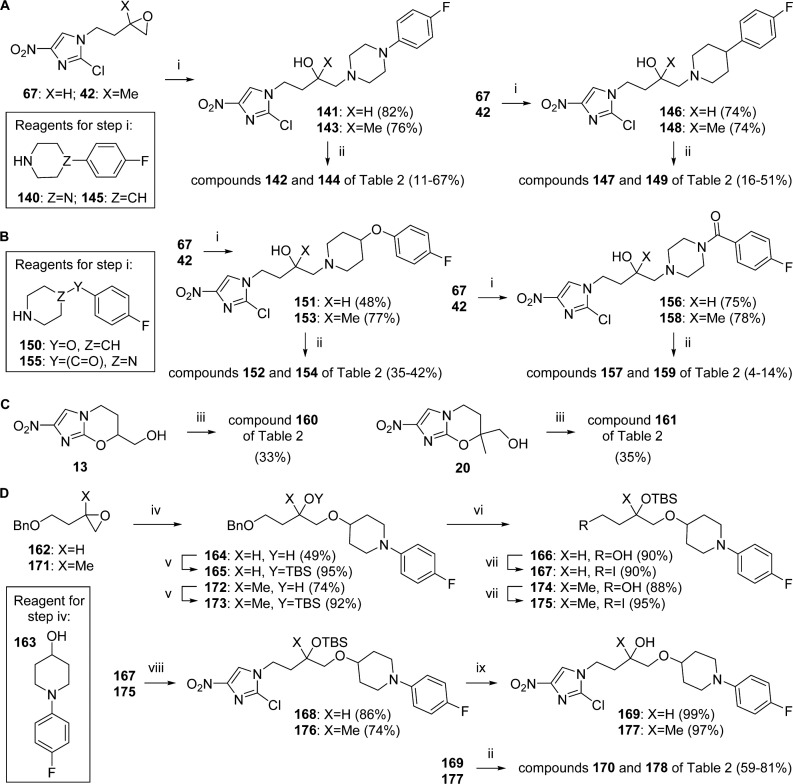
^a^Reagents and conditions: (i) amine (**140**, **145**, **150**, or **155**), MEK (or DME), 70–85 °C, 16–84 h; (ii) NaH, DMF, 40–60 °C or 0–20 °C (for **170**)or20–39 °C(for **178**), 1.5–5 h; (iii) triphosgene, Et_3_N, THF, 20 °C, 30 min, then **140**, THF, 20 °C, 2 h; (iv) **163**, NaH, DMF, 70 °C, 14–28 h; (v) TBSOTf, 2,6-lutidine, CH_2_Cl_2_, 0–20 °C, 1–2.5 d; (vi) H_2_ (60 psi), 10% Pd–C, EtOH, EtOAc, 20 °C, 45–51 h; (vii) I_2_, PPh_3_, imidazole, CH_2_Cl_2_, 20 °C, 17–19 h; (viii) 2-chloro-4-nitroimidazole, K_2_CO_3_, DMF, 63–75 °C, 70–72 h; (ix) TBAF, THF, 20 °C, 5–25 h.

Scheme 5 outlines the methods used to obtain compounds **142**, **144**, **147**, **149**, **152**, **154**, **157**, **159–161**, **170**, and **178**, whose side chains contained either piperazine or piperidine linked to an aryl group. Ring opening of epoxides **67** and **42** with the known or commercial amines **140**, **145**, **150**,^34^ and **155** easily generated the expected *β*-amino alcohols in high yield (Scheme 5A,B). These alcohols could be ring closed to the final products with sodium hydride upon mild heating, albeit yields for the 7-methyl analogues were generally significantly lower, in part due to greater purification difficulties. Chloroformylation of alcohols **13** and **20** (triphosgene/Et_3_N) and in situ reaction with arylpiperazine **140** also led to the *O*-carbamates **160** and **161** in only modest yield (33–35%; Scheme 5C) on account of similar purification issues; the isolation of alkyl chloride and diethyl carbamate derivatives under the same reaction conditions has been reported recently.^35^ Lastly, synthesis of the two *O*-linked arylpiperidines, **170** and **178**, was eventually achieved in each case via a lengthy seven-step route (Scheme 5D), after the failure of a more direct plan (ring opening of epoxide **67** with piperidinol **163**^36^ in the presence of erbium triflate^37^). Here, piperidinol **163**^36^ was first sourced in three steps by Buchwald amination of 1-bromo-4-fluorobenzene with 1,4-dioxa-8-azaspiro[4.5]decane,^38^ ketal hydrolysis, and reduction (NaBH_4_). Reaction of epoxides **162**^39^ and **171**^39^ with **163** (NaH, DMF, 70 °C) and TBS protection of the liberated hydroxyls provided the desired ethers **165** and **173** in good yield (47–68% overall). Successive benzyl removal (via hydrogenolysis), iodination, and alkylation of 2-chloro-4-nitroimidazole (**40**) then gave the TBS-protected adducts, **168** and **176**, which were readily desilylated (TBAF) and ring closed to furnish the final targets.

## ■ RESULTS AND DISCUSSION

The structures and in vitro antiparasitic and antitubercular potencies of 75 novel 7-substituted 2-nitroimidazooxazine derivatives prepared in two collaborative projects are provided in Tables 1 and 2. While compounds **14**, **21–23**, **25**, **27–29**, **34**, **38**, **39**, **44**, **45**, **47**, **49**, and **52–54** were initially designed and evaluated for TB, for clarity purposes, we will focus the discussion first on the more recent VL work with DND*i*. Here, new synthesis was directed at the optimization of solubility, efficacy, and safety, primarily through the incorporation of heterocycles to reduce compound lipophilicity^11^ (estimated by CLogP data derived from ACD LogP/LogD software, version 12.0; Advanced Chemistry Development Inc., Toronto, Canada). Kinetic aqueous solubility measurements were conducted on dry powder forms of particular examples that were being considered for further evaluation. Target compounds were initially screened only once against *Leishmania donovani* (*L. don*) using a mouse macrophage-based luciferase assay conducted at the Central Drug Research Institute (CDRI, India).^10^ Nevertheless, to gain a clearer understanding of the SAR (in view of some unexpected in vivo outcomes), the entire set was finally re-evaluated at the University of Antwerp (LMPH) in replicate assays against three protozoan parasites: *Leishmania infantum* (*L. inf*), *Trypanosoma cruzi*, and *Trypanosoma brucei*.^40^ Assessments of cytotoxicity were concurrently conducted on both human lung fibroblasts (MRC-5 cells; the host for *T. cruzi*) and primary peritoneal mouse macrophages (the host for *L. inf*), which revealed that the compounds were generally nontoxic (MRC-5 IC_50_s > 55 *μ*M except for **117**: IC_50_ 35 *μ*M), as confirmed for TB (VERO assay^41^ IC_50_s > 128 *μ*M for 71 of 72 compounds).

**Table 1:** In Vitro Antiparasitic and Antitubercular Activities and Calculated Lipophilicities of 7-Substituted 2-Nitroimidazooxazines 
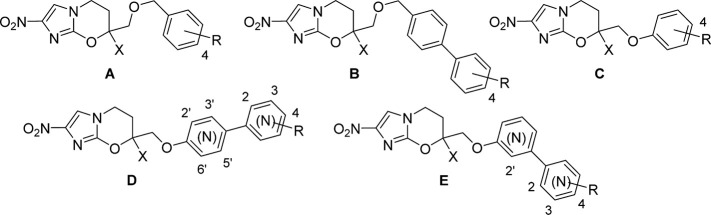

compd	form	X	aza	R	CLogP	IC_50_^*a,b*^ (*μ*M)				MIC^*c,b*^ (*μ*M)	
						*L. don*	*L. inf*	*T. cruzi*	MRC-5	MABA	LORA
**4**^*d*^					3.47	0.03	0.17	2.6	>64	0.046	5.9
**14**	A	H		4-OCF_3_	3.30	0.03	0.12	1.2	>64	1.0	7.5
**21**	A	Me		4-OCF_3_	3.68	0.31	0.30	0.75	>64	0.55	3.3
**22**	A	H		4-OBn	3.55	0.05				0.46	3.0
**23**	A	Me		4-OBn	3.93	0.28				0.20	1.4
**25**	B	H		4-F	4.21	0.02	0.17	0.53	>64	0.08	1.3
**27**	B	Me		4-F	4.59	0.22	1.8	0.84	>64	0.085	0.61
**28**	B	H		4-OCF_3_	5.14	0.19	0.40	2.1	>64	0.055	1.5
**29**	B	Me		4-OCF_3_	5.52	1.1	1.1	0.54	>64	0.093	1.4
**34**	B^*e*^	Me		4-OCF_3_	5.52	0.24	1.3	0.57	>64	0.063	1.1
**38**	B^*f*^	Me		4-OCF_3_	5.52	1.3	1.3	0.77	>64	0.74	6.8
**39**	C	H		4-OCF_3_	3.37	0.04	0.047	0.061	>64	5.2	4.7
**44**	C	Me		4-OCF_3_	3.75	0.10	0.13	0.14	>64	0.94	6.8
**45**	C	H		4-OBn	3.62	0.02				>128	>128
**47**	C	Me		4-OBn	4.00	0.12				0.44	>128
**49**	D	H		4-F	4.11	0.01	0.47	0.063	>64	0.18	>128
**52**	D	Me		4-F	4.49	0.20	0.34	0.35	>64	0.085	1.4
**53**	D	H		4-OCF_3_	5.03	0.06	0.28	0.24	>64	0.08	0.73
**54**	D	Me		4-OCF_3_	5.41	0.52	0.36	0.72	>64	0.11	1.3
Phenylpyridines											
**55**	D	H	3	4-F	2.94	>10	0.083	0.027	>64	0.24	3.5
**56**	D	Me	3	4-F	3.32	0.12	0.16	0.30	>64	0.29	2.7
**59**	D	H	2′	4-F	3.66	0.01	0.050	0.027	>64	0.025	<0.25
**61**	D	Me	2′	4-F	4.04	0.09	0.097	0.23	>64	0.17	1.0
**62**	D	H	2′	2,4-diF	3.65	0.07	0.037	0.030	>64	0.10	2.4
**63**	D	Me	2′	2,4-diF	4.03		0.11	0.21	>64	0.089	2.5
**64**	D	H	2′	4-OCF_3_	4.58	0.05	0.35	0.12	>64	0.027	0.47
**65**	D	Me	2′	4-OCF_3_	4.96	0.26	3.8	1.0	>64	0.13	5.3
**71**	D	H	3′	4-F	3.03	0.06	0.093	0.27	>64	0.23	2.4
**79**	D^*e*^	H	3′	4-F	3.03	(0.03)^*g*^	0.080	0.35	>64	0.11	3.2
**87**	D^*f*^	H	3′	4-F	3.03	(0.08)^*g*^	0.22	0.29	>64	1.1	3.9
**90**	D	Me	3′	4-F	3.41	0.65	0.59	0.26	>64	0.35	3.9
**91**	D	H	3′	2,4-diF	3.03	0.02	0.030	0.13	>64	0.36	8.9
**92**	D	Me	3′	2,4-diF	3.41	0.31	0.17	0.27	>64	0.40	4.9
**93**	D	H	3′	4-OCF_3_	3.96	0.05	0.12	0.17	>64	0.032	0.86
**94**	D^*e*^	H	3′	4-OCF_3_	3.96		0.11	0.26	>64	0.024	1.5
**95**	D^*f*^	H	3′	4-OCF_3_	3.96		0.13	0.13	>64	0.34	1.6
**96**	D	Me	3′	4-OCF_3_	4.34	0.65	4.0	0.25	>64	0.05	0.88
**99**	E	H	2′	4-F	3.55	0.31	0.14	0.16	59	1.7	3.0
**101**	E	Me	2′	4-F	3.93		0.18	0.35	>64	0.94	5.0
**102**	E	H	2′	2,4-diF	3.55	0.25	4.0	0.23	>64	>128	>128
**103**	E	Me	2′	2,4-diF	3.93		0.24	0.22	>64	0.69	4.8
Bipyridines											
**105**	D	H	2′,2	4-F	2.55	0.06	50	0.34	>64	0.15	11
**106**	D	Me	2′,2	4-F	2.93	0.15	0.34	0.25	>64	0.40	9.5
**107**	D	H	2′,3	4-F	2.49	0.27	47	0.54	>64	0.074	15
**108**	D	Me	2′,3	4-F	2.87	0.22	0.40	0.82	>64	1.9	6.2
**109**	D	H	2′,3	2,4-diF	2.60	>10	0.19	0.29	>64	0.25	4.2
Bipyridines											
**110**	D	Me	2′,3	2,4-diF	2.98		0.20	0.43	>64	1.0	6.6
**111**	D	H	2′,3	4-CF3	2.89	0.13	2.5	0.68	>64	0.09	2.5
**112**	D	H	3′,3	4-F	1.87	0.09	>64	0.55	>64	2.3	21
**113**	D	Me	3′,3	4-F	2.25	0.09	0.67	0.57	>64	2.7	43
**114**	D	H	3′,3	2,4-diF	1.98	0.08	52	0.35	>64	1.2	41
**115**	D	Me	3′,3	2,4-diF	2.36	0.11	0.28	0.38	>64	0.94	18
**116**	D	H	3′,3	4-CF3	2.61	0.07	7.3	0.46	>64	0.12	58
**117**	E	H	2′,3	4-F	2.38	>10	44	0.13	35	>128	>128
**118**	E	Me	2′,3	4-F	2.76		1.1	0.52	>64	11	7.8
**119**	E	H	2′,3	2,4-diF	2.50	0.25	6.7	0.13	>64	4.3	10
**120**	E	Me	2′,3	2,4-diF	2.88		0.59	0.49	>64	9.3	7.9
Other Heterobiaryls											
**123**	D	H	2′,3′	4-F	2.73	0.07	8.4	0.67	>64	0.35	23
**126**	D	H	2′,5′	4-F	3.09	0.15	0.71	4.7	>64	0.15	>128
**129**	D	H	2′,6′	4-F	2.58	0.10	0.29	0.17	>64	0.04	1.7
**131**	D	Me	2′,6′	4-F	2.96	0.32	0.29	0.43	57	0.44	6.3
**132**	D	H	2′,6′,3	4-F	1.41	0.53	>64	3.3	>64	0.24	21
**133**	D	Me	2′,6′,3	4-F	1.79	1.3	6.1	2.9	>64	23	54
**139**	D	H	3′,5′	4-F	2.79	0.07	0.21	0.26	62	0.27	20

a ICso values for inhibition of the growth of *Leishmania donovani* and *Leishmania infantum* (in mouse macrophages) and *Trypanosoma cruzi* (on MRC-5 cells), or for cytotoxicity toward human lung fibroblasts (MRC-5 cells).

b Each value (except the single test L. *don* data) is the mean of at least two independent determinations. For complete results (mean ± SD) please refer to the Supporting Information.

c Minimum inhibitory concentration against *M. tb*, determined under aerobic (MABA)^41^ or hypoxic (LORA)^56^ conditions.

d Data from ref 11.

e (R)-Enantiomer.

f (S)-Enantiomer.

g LMPH data (mean of 2 values).

**Table 2:** In Vitro Antiparasitic and Antitubercular Activities and Calculated Lipophilicities of Cyclic Amine-Based Analogues 
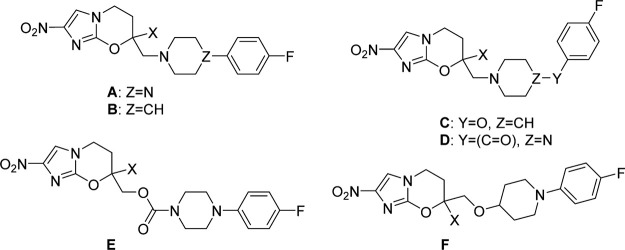

compd	form	X	CLogP	IC_50_^*a,b*^ (*μ*M)				MIC^*c,b*^ (*μ*M)	
				*L. don*	*L. inf*	*T. cruzi*	MRC-5	MABA	LORA
**142**	A	H	2.16	0.19	2.3	1.7	>64	1.8	9.9
**144**	A	Me	2.54	0.70	0.73	1.3	>64	0.34	6.8
**147**	B	H	3.36	0.88	0.87	2.9	>64	2.1	11
**149**	B	Me	3.74	1.0	0.32	0.87	>64	0.22	8.3
**152**	C	H	2.84	0.45	0.84	2.4	>64	2.0	22
**154**	C	Me	3.22	0.22	0.32	0.59	>64	0.23	48
**157**	D	H	1.17	4.8	45	11	>64	46	>128
**159**	D	Me	1.55	2.8	6.5	2.8	>64	93	>128
**160**	E	H	2.42	>100	>64	1.5	>64	2.5	22
**161**	E	Me	2.80	0.29	0.72	3.9	>64	3.4	8.4
**170**	F	H	3.50		0.24	0.22	>64	0.78	20
**178**	F	Me	3.88		0.35	0.51	>64	0.37	19

a IC50 values for inhibition of the growth of *Leishmania donovani* and *Leishmania infantum* (in mouse macrophages) and *Trypanosoma cruzi* (on MRC-5 cells), or for cytotoxicity toward human lung fibroblasts (MRC-5 cells).

b Each value (except the single test L. *don* data) is the mean of at least two independent determinations. For complete results (mean ± SD) please refer to the Supporting Information.

c Minimum inhibitory concentration against *M. tb*, determined under aerobic (MABA)^41^ or hypoxic (LORA)^56^ conditions.

**Early Hit to Lead Assessments for VL**. Through an agreement between TB Alliance and DND*i*, 58 nitroimidazole derivatives were screened against *L. don* at the Swiss Tropical Institute. All five 7-substituted oxazines (including **22**, **23**, and **28**) demonstrated excellent potencies in the in vitro mouse macrophage assay (IC_50_s 0.065–0.17 *μ*M, similar to racemic **4**), prompting the inclusion of **28** alongside *rac*-**4** (and another oxazole^11^) in a proof-of-concept in vivo assessment at the London School of Hygiene and Tropical Medicine (LSHTM). However, the level of activity observed for **28** in this *L. don* mouse model (49% inhibition at 50 mg/kg, dosing po daily for 5 d; Table 3) was not notable in comparison to the results for *rac*-**4** (99% at 6.25 mg/kg),^11^ suggesting that further optimization of the side chain would be necessary. Indeed, while **28** showed good stability on exposure to mouse liver microsomes (MLM: 75% remaining after 1 h, Table 3) and gave a mouse pharmacokinetic (PK) profile comparable to *rac*-**4** (Table 4 and Supporting Information, Figures S1 and S2), it was very hydrophobic (CLogP 5.14) and displayed poor solubility (∼58 ng/mL at pH 7, Table 3; 62-fold lower than for *rac*-**4**^11^). Such compounds typically exhibit high levels of plasma protein binding (PPB), which can limit efficacy.^42^ We have also observed that increased linker flexibility can be detrimental to in vivo activity.^17,43^

**Table 3:** Aqueous Solubility, Microsomal Stability, and in Vivo Antileishmanial Efficacy Data for Selected Analogues

compd	aq solubility^*a*^ (*μ*g/mL)		microsomal stability^*b*^ [% remaining at 1 (0.5) h]			in vivo efficacy against *L. don* (mouse) (% inhibition at dose in mg/kg)^*c*^					
	pH 7	pH 1	H	M	Ham	50	25	12.5	6.25	3.13	1.56
**4**^*d*^	2.4		(92)	(89)	18 (54)			>99	>99	83	49
**14**	4.8		57	10							
**28**	0.058		73	75	46	49					
**29**	0.39		85	77							
**34**			86	79							
**38**			86	59							
**39**	4.0		85 (96)	57 (70)	(23)		87				
**44**	2.3		58 (86)	50 (61)	2.1 (16)		100	100	83	25	
**49**	0.055		(88)	(75)	(45)		>99				
**53**	0.13		(97)	(94)	(94)		65				
**54**	2.6		(89)	(85)	(81)		30				
**55**	0.36										
**59**	0.13	2.8	41	43 (60)	12 (16)	67					
**61**	0.69	13		(33)	(2.6)						
**62**	0.13	1.5	25	19	2.7						
**71**	0.32	164	44	34 (63)	14 (52)	100	98	76	59		
**79**	0.45	237	58	69	34				93		
**87**	0.47	234	63	41	5				85		
**90**	0.19	74	50	28	32	41					
**91**	0.72	221	45	31	8.6	91					
**93**	0.15	92	53	41	37	>99			>99	97	50
**94**	0.37	110	50	53						>99	84
**95**	0.40	141	52	46						52	57
**99**	0.027	0.56									
** 107**	0.87			(81)	(51)						
** 108**	4.5			(68)	(39)						
** 111**	0.36			(72)	(70)						
** 112**	3.9		(93)	(87)	(70)	44					
** 113**	2.3			(82)	(43)						
** 116**	0.30			(82)	(81)						
** 129**	1.8		61	56	13	85					
** 139**	0.59										
** 142**	10	14900	85	77	15	55					
** 152**	49	21100	57	58	0						
** 170**	6.1	34300	33	17	0.7	45					
** 178**	2.2	9970	11	1.2	0.2						

a Kinetic solubility (*μ*g/mL) in water (pH 7) or 0.1 M HCl (pH 1) at 20 °C, determined by HPLC (see the Experimental Section, Method A).

b Pooled human (H), CD-1 mouse (M), or hamster (Ham) liver microsomes; data in parentheses are the percentage parent compound remaining following a 30 min incubation.

c Dosing was orally, once daily for 5 days consecutively; data are the mean percentage reduction of parasite burden in the liver.

d Data from ref 11

**Table 4:** Pharmacokinetic Parameters for Selected Compounds in Various Species

compd	intravenous (1–2 mg/kg)^*a*^						oral (5–40 mg/kg)^*a*^					
	C_0_ (*μ*g/mL)	CL (mL/min/kg)	Vdss (L/kg)	*t*_1/2_ (h)	AUC_last_^*b*^ (*μ*g·;h/mL)	C_max_ (*μ*g/mL)	T_max_ (h)	*t*_1/2_ (h)	AUC_last_^*b*^ (*μ*·h/mL)	*F*^*c*^(%)
					Mice					
*rac-**4**^*d*^*	0.88	9.5	1.7	2.2	1.69	4.1	4.0		33.5	79
**28**						3.3	8.0	5.2	47.1^*e*^	
**34**						2.0	6.0	8.1	31.9^*e*^	
**39**	0.36	48	3.2	1.1	0.341	1.3	0.5		3.86	45
**44**	0.79	12	2.5	2.8	1.31	1.4	3.0		11.5	35
**49**	2.9	0.52	0.40	6.7	31.6	4.2	6.0		84.7	11
**53**	0.66	1.3	1.9	17	11.2	14	8.0		376	100
**54**	0.43	2.3	5.0	27	5.26	0.79	10		22.7	17
**71**						13	4.0	3.4	112	
**112**	14	0.70	0.12	2.1	24.7	14	2.0		95.3	31
					Rats					
**71**	1.3	3.9	0.94	3.0	4.36	0.49	3.3	6.7	4.27	22
**79**	1.5	6.3	0.86	1.7	2.65	0.79	3.3	3.1	4.08	34
**87**	1.1	5.5	1.4	3.2	3.14	0.71	3.3	3.7	5.64	41
					Hamsters					
**71**	2.5	11	1.0	1.2	3.12	0.94	2.7	4.7	4.83	26
**79**						1.4	2.0	4.2	6.82	
**87**						0.73	2.7	10	3.68	

a The intravenous dose was 1 mg/kg for mice and rats and 2 mg/kg for hamsters. The oral dose in mice was 25 mg/kg, except for **28** and **34** (40 mg/kg) and **112** (12.5 mg/kg); in other species, the oral doses were 5 mg/kg (rats) or 12.5 mg/kg (hamsters).

b Area under the curve calculated to the last time point (10, 24, or 48 h).

c Oral bioavailability, determined using dose normalized AUC_last_ values.

d Data for racemic **4** from ref 11.

e Extrapolated AUC_inf_ value.

While these mouse studies were being conducted, a further 30 7-substituted oxazine derivatives were screened against *L. don* in the luciferase assay at CDRI.^10^ On the basis of the single IC_50_ data obtained for **14**, **21–23**, **25**, **27–29**, **34**, **38**, **39**, **44**, **45**, **47**, **49**, and **52–54** (Table 1), several preliminary SAR conclusions were drawn: (1) the 7-H series was generally 5–10-fold more potent than the 7-methyl series; (2) 4-trifluoromethoxy and 4-benzyloxy substituents (forms A and C) provided equivalent potency; (3) for biaryl analogues (forms B and D), 4-fluoro was preferred over 4-trifluoromethoxy as the final ring substituent (as observed^11^ in the 6-nitroimidazooxazole series); (4) a shorter linker (forms C and D) was preferred in the majority of cases. Thus, the most active analogues appeared to be **14**, **22**, **25**, **39**, **45**, **49**, and **53** (IC_50_s 0.01–0.06 *μ*M, similar to **4**). However, benzyl ether **14** did not display suitable metabolic stability (10% parent left after 1 h with MLM; Table 3), while evidence from the 6-substituted oxazine series^17^ for the more rapid metabolism of benzyloxybenzyl analogues dissuaded further testing of **22** and **45**. Moreover, following the disappointing results with **28**, we were not optimistic of good in vivo efficacy with close analogue **25** despite its improved potency. Therefore, we elected to initially investigate **39**, **49**, and **53** as potential leads, together with two counterparts from the 7-methyl series, **44** and **54**, to enable a head-to-head comparison.

**Figure 3:**
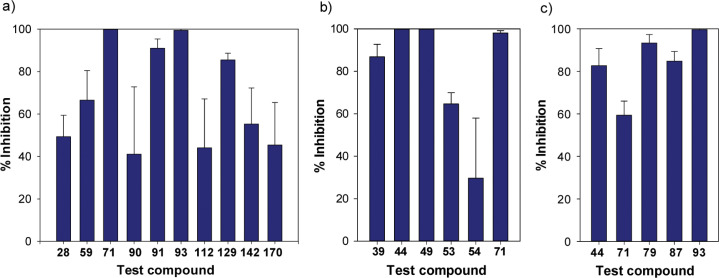
Comparative in vivo efficacy against *L. don* in the mouse model: (a) 50 mg/kg, (b) 25 mg/kg, and (c) 6.25 mg/kg.

The selected compounds were advanced to parallel mouse PK profiling and efficacy studies in the mouse VL model. Encouragingly, both phenyl ether **44** (the direct analogue of *rac*-**4**) and biphenyl congener **49** showed excellent efficacy at 25 mg/kg (99.9–100% inhibition; Table 3 and Figure 3b). Surprisingly, the more potent 7-H counterpart of **44** (**39**) was slightly less active in this assay (87% inhibition), mimicking findings for the 2-H equivalent of *rac*-**4**.^11^ Moreover, the biphenyl derivatives of **39** and **44** (**53** and **54**) were also less impressive than **49** (65% and 30% inhibition, respectively). However, while these latter results appeared to track well with the single determination *L. don* data, they did not seem to line up with the almost equivalent mean potencies vs *L. inf* (Table 1). The findings also appeared to conflict with the kinetic solubility and microsomal stability data (Table 3), where **49** was as poorly soluble as **28** (55 vs 58 ng/mL), but the more stable analogue **54** (85 vs 75% in MLM) was 45-fold more soluble than **28** (2.6 *μ*g/mL). Solubility is discussed further in the next section.

Analysis of the mouse PK data (Table 4) provided greater insight, revealing that **39** had a 4-fold higher rate of clearance than its 7-methyl derivative **44** (48 vs 12 mL/min/kg), resulting in a short half-life (1.1 h vs 2.8 h for **44**) and quite poor oral exposure (see the Supporting Information, Figure S1). Interestingly, with iv administration, the PK profiles for **44** and *rac*-**4** were fairly similar, but **44** did not perform as well under oral dosing, with rather modest absorption (*C*_max_ 1.4 *μ*g/mL, 3-fold less than for *rac*-**4**) contributing to reduced exposure and moderate oral bioavailability (35% vs 79%). The oral parameters for compound **54** were also mediocre (poor *C*_max_ of 0.79 *μ*g/mL and low oral bioavailability of 17% offsetting its lengthy 27 h half-life), potentially explaining its inferior efficacy in the mouse VL model. However, the findings for **49** and **53** were more puzzling, with the less efficacious **53** demonstrating greater oral exposure (see the Supporting Information, Figure S1), superior oral bioavailability (100% vs 11% for **49**), and an extended half-life (17 h vs 6.7 h for **49**). Nevertheless, like **28**, **53** was particularly hydrophobic (CLogP 5.03), so high PPB may be a major issue limiting its efficacy.^42^ We have previously observed that PK data is not always correctly predictive of in vivo efficacy ranking.^43^

These promising results prompted further appraisal of the most active compounds, **44** and **49**. In the mouse VL model, **44** provided robust dose–response data (Table 3), giving an ED_50_ value of 4.2 mg/kg (cf. 3.0 mg/kg for *rac*-**4**^11^). Unfortunately, additional studies of **49** in this model (using material prepared elsewhere) were unable to replicate the original result; we postulate that this discrepancy may be due in part to the extremely poor aqueous solubility and inadequate oral bioavailability of this compound, rendering oral suspension formulations particularly sensitive to particle size. Nevertheless, the optimal in vivo assay for assessing the efficacy of test compounds against VL is the chronic infection hamster model, which better reproduces the clinical pathology of human disease.^44^ In the *L. don* hamster model at CDRI, leads **44** and **49** were almost equally effective at 50 mg/kg, with 5 days of oral dosing leading to 53% and 51% inhibition of parasite infection in the spleen, whereas *rac*-**4** gave 86% inhibition under the same dose regimen.^11^

A significant factor in the suboptimal activity of **44** in the hamster model was thought to be its exceptionally rapid metabolism in this species, as revealed by the hamster microsomal stability data (only 16% remaining after 0.5 h vs 49% for *rac*-**4**^11^). Therefore, **44** was later reassessed in the *L. inf* early curative hamster model at LMPH, comparing a twice-daily dose regimen (25 mg/kg b.i.d.) with a once daily dose of 50 mg/kg. The results (Table 5) slightly favored the twice daily regimen for all three target organs; hence, this protocol became standard for most test compounds. However, unlike **4**, **44** was not curative at this dose level. Another liability with **44** was its greater inhibition of the hERG channel (IC_50_ 3.8 vs 10.5 *μ*M for **4**), with IC_50_ values in excess of 10 *μ*M required to minimize QT prolongation risk.^45^ Hence, as lead compounds for VL, **44** and **49** fulfilled many suggested criteria^46^ but still had key deficiencies, reflecting their origin as screening hits in a scarcely studied new class.

**Table 5:** In Vivo Efficacy Data for Selected Lead Compounds in the *L. inf* Hamster Model

compd	dose regimen^*a*^ (mg/kg)	% inhibition in target organs^*b*^ liver spleen bone marrow		
**1**	40, qd	92.6	99.5	89.0
**4**^*c*^	25, qd	100	99.9	99.7
	12.5, qd	99.0	98.7	94.0
**44**	25, b.i.d.	92.2	91.1	82.5
	50, qd	88.6	89.5	73.0
**71**	25, b.i.d.	99.9	99.4	99.6
	12.5, b.i.d.	99.9	99.5	99.4
	6.25, b.i.d.	98.0	95.7	96.3
	3.13, b.i.d.	68.3	69.8	64.5
	12.5, qd	95.2	87.5	92.8
**79**	12.5, b.i.d.	99.5	99.4	96.8
	6.25, b.i.d.	91.0	91.6	73.3
**87**	12.5, b.i.d.	88.2	80.8	82.3
	6.25, b.i.d.	53.1	46.7	35.0

a All compounds were dosed orally, once or twice daily for five days consecutively.

b Data are the mean percentage reduction of parasite burden in target organs.

c Data from ref 11.

**SAR of 7-Substituted 2-Nitroimidazooxazines for VL**. Following the identification of **4** as a preferred drug candidate and the discovery of **44** and **49** as unoptimized new leads, a backup program was launched to develop second-generation agents for VL having better solubility, PK–PD, and safety profile.^11^ Because of the inferior profile of **44** in comparison to **4** in several key areas, we elected to center our synthetic strategy mainly on bicyclic side chains, employing heterocycles to modulate lipophilicity and solubility. Six-membered ring nitrogen-containing variants were preferred due to their greater metabolic stability;^47^ *ortho*-substitution of aryl groups and *meta*-linkage of rings were also investigated as additional options to increase solubility.^48^ Recognizing that few orally active registered drugs have solubility values below 1 *μ*M at pH 7.4 (the pH of blood),^49^ we aspired to achieve at least 10-fold higher than this for the best compounds.^46^ We also aimed to exploit the low pH of gastric fluid (∼1–2) to improve dissolution and oral absorption of analogues containing pyridine and other bases.^50^ Hence, we set a minimum solubility requirement for the preferred final candidate of being noninferior to delamanid (**5**) (0.31 *μ*g/mL at pH 7 and 116 *μ*g/mL at pH 1),^11^ an approved TB drug in Europe and Japan.^12^

On the basis of the wider in vitro screening results, it was apparent that the 7-substituted oxazines could not be used for African trypanosomiasis (*T. brucei* IC_50_s mostly >64 *μ*M, none <1 *μ*M; see Supporting Information, Tables S1 and S2). However, unlike the 6-nitroimidazooxazoles, this new oxazine class generally showed interesting potencies against *T. cruzi* (IC_50_s 0.03–1 *μ*M), suggesting the possibility of dual utility to treat both VL and Chagas disease. Further analysis of data for the 65 racemic compounds tested indicated a modest trend for the best VL leads to have high potencies against *T. cruzi* (see Supporting Information, Figure S4). Hence, for simplicity, we will focus this part of the SAR discussion entirely on the intended primary application (VL), emphasizing the key *L. inf* results.

To begin with, a reanalysis of the initial data set (up to and including **54**; Table 1) confirmed weak trends on *L. inf* for the 7-H analogues to be more potent and a shorter linker length to be preferred (e.g., **39**: IC_50_ 0.047 *μ*M), but there was no consistent preference for 4-fluoro as the terminal ring substituent. Nevertheless, in view of the better in vivo efficacy of **49** and similarly substituted nitroimidazooxazoles,^11^ we retained this latter design element in the majority of cases. Thus, compounds **55** and **56** first investigated the effect of replacing the second phenyl ring of **49** by pyridine (ΔCLogP–1.2 units). Pleasingly, this led to a 2–6-fold potency increase, with **55** (IC_50_ 0.083 *μ*M) also being 6.5-fold more soluble than **49** (0.36 vs 0.055 *μ*g/mL, Table 3). Exchange of the first phenyl ring by 2-pyridine (**59** and **61**; ΔCLogP –0.5 units) resulted in even better activity (**59**:IC_50_ 0.050 *μ*M), and in this case solubility values were ∼20-fold higher at low pH (2.8–13 *μ*g/mL; calcd p*K*_a_ 2.83) although still rather modest. Therefore, we examined the addition of an *ortho* fluorine in the phenyl ring (**62** and **63**) in an attempt to break up the planarity.^48^ However, while this change was well tolerated, there was no improvement in solubility and microsomal stability was reduced (19% vs 43% in MLM for **62** vs **59**, Table 3). In an alternative approach, we tried *meta-*linkage of the rings (**99** and **101–103**), but although the activity was generally acceptable, this led to inferior solubility (**99**: 27 ng/mL).

Turning instead to 3-pyridine as a first ring, potency was maximized by 2,4-difluorophenyl substitution (**91**: IC_50_ 0.030 *μ*M) although the 4-fluoro and 4-trifluoromethoxy (7-H) analogues (**71** and **93**) were also useful (IC_50_s 0.093 and 0.12 *μ*M, respectively). Importantly, aqueous solubility values were up to 13-fold better than for **49** at neutral pH (**91**: 0.72 *μ*g/mL) and 3 orders of magnitude better at low pH (**91**: 221 *μ*g/mL). This was consistent with a greater lipophilicity reduction for the 3-pyridine (ΔCLogP –1.1 units) and a slightly higher basicity (e.g., **71**: calcd p*K*_a_ 3.76). Final assessment of the enantiomers of two examples, **71** and **93**, identified the *R* forms (**79** and **94**) as slightly preferred for both potency and microsomal stability (particularly in the case of **79**).

**Figure 4:**
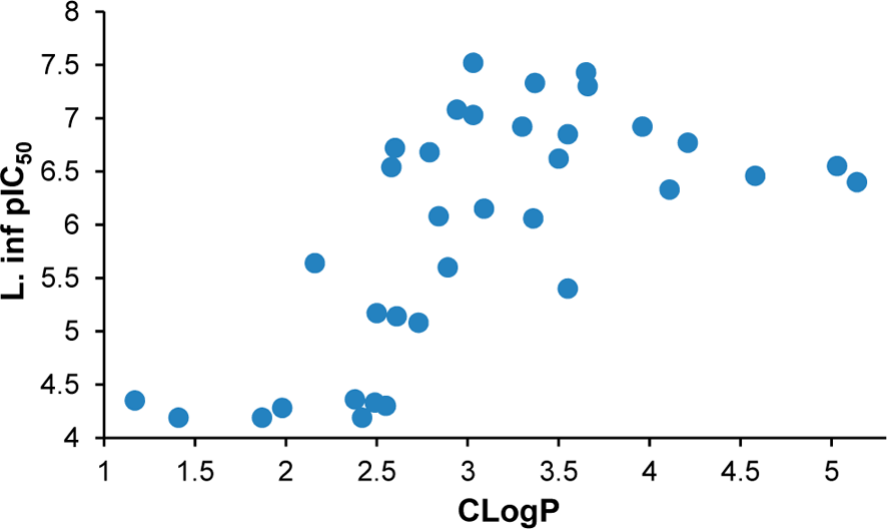
Effect of lipophilicity on potency against *L. inf* for 35 racemic 7-H analogues.

In view of the promising results with phenylpyridines, we elected to investigate the more hydrophilic bipyridines (**105–120**). Most of these showed interesting potencies in the initial *L. don* screen and further assessments had identified **108**, **112**, and **113** as being of potential interest based on their improved solubilities in comparison to **49** (2.3–4.5 vs 0.055 *μ*g/mL). However, on retesting, almost all of the 7-H compounds displayed markedly inferior utility against *L. inf* (IC_50_s 2.5 to >64 *μ*M) while the 7-methyl bipyridines retained moderate potencies (IC_50_s0.20–1.1 *μ*M). It is intriguing to speculate that this might indicate a “minimum lipophilicity” requirement for activity (e.g., CLogP ∼ 2.5) because a similar pattern was noted for all of the more hydrophilic analogues (see analysis of racemic 7-H data set, Figure 4). Another strategy for heterobiaryl analogues of **49** was to exchange the first phenyl ring with pyridazine, pyrazine, or pyrimidine (**123**, **126**, **129**, **131–133**, and **139**). Of these, pyrimidine (**129**, **131**, and **139**) provided the best activity (IC_50_s 0.21–0.29 *μ*M), although combining this with a pyridine ring (**132**, **133**) led to a dramatic loss of potency (21- to >220-fold). Overall, pyrimidine **129** had the best aqueous solubility (1.8 *μ*g/mL; 33-fold better than **49**) along with acceptable metabolic stability.

More structurally diverse targets (**142**, **144**, **147**, **149**, **152**, **154**, **157**, **159–161**, **170**, and **178**; Table 2) were designed on the premise that arylated cyclic amines can be effective bioisosteres for biphenyls, thus facilitating substantial boosts in solubility.^51,52^ Several side chains of this type have previously shown promise for TB and/or VL,^11,18^ including in the recent development of antileishmanial aminopyrazole ureas.^53^ It was initially encouraging to see four examples (arylpiperazine **142**, aryloxypiperidines **152** and **154**, and arylpiperazine carbamate **161**) exhibiting reasonable potencies in the *L. don* screen (IC_50_s 0.19–0.45 *μ*M), with **142** and **152** displaying a markedly better solubility profile than **49** (10–49 *μ*g/mL at pH 7, 15–21 mg/mL at low pH). However, the *L. inf* data did not fully match the *L. don* results; instead, the 7-methyl analogues were clearly favored over the 7-H compounds (by 3–7-fold), and the more lipophilic piperidines **149** and **154** were superior (IC_50_s 0.32 *μ*M). The hydrophilic benzoylpiperazines **157** and **159** were particularly poor in both assays, as was the 7-H arylpiperazine carbamate **160**. In view of these SAR findings, two O-linked phenylpiperidines (**170** and **178**) were subsequently designed as structurally closer mimetics for the O-linked biphenyl **49**. Gratifyingly, **170** demonstrated both good potency (IC_50_ 0.24 *μ*M) and much better solubility than **49** (6.1 *μ*g/mL at pH 7, 34 mg/mL at pH 1), albeit the microsomal stability of this compound (17–33% in MLM and HLM) was regarded as quite marginal.

Integration of the initial *L. don* data with the kinetic solubility and microsomal stability results led to the selection of nine new racemic analogues of **49** for testing in the *L. don* mouse model (dosing at 50 mg/kg for 5 d; Table 3 and Figure 3a). Encouragingly, a first experiment on 3-pyridine derivative **71** (4-FPh) yielded a 100% parasite clearance from the liver in all mice. Following this, 4-trifluoromethoxy congener **93** was found to be equally efficacious (99.5%), whereas the 2,4-difluoro example **91** was slightly less effective (91% inhibition). However, the less potent 7-methyl derivative of **71** (**90**) and the more potent 2-pyridine analogue **59** were only moderately active (41% and 67%, respectively); it is possible that the higher crystallinity (larger particle size) of **59** may have contributed to poor oral bioavailability.^42,48^ Two more soluble heterobiaryl analogues, bipyridine **112** and phenylpyrimidine **129**, also displayed lower efficacy (44% and 85% inhibition); oral PK data on **112** (Table 4 and Supporting Information, Figure S2) were comparable to those of **71**, so this may be a potency issue (as suggested by the disparate *L. inf* and *L. don* IC_50_s of >64 vs 0.09 *μ*M). Finally, the inferior in vivo outcomes for two potential bioisosteres of **49**, phenylpiperazine **142** (55%) and O-linked phenylpiperidine **170** (45%), may be attributed to either weaker in vitro activity on retesting (for **142**: *L. inf* IC_50_ 2.3 *μ*M) or more rapid metabolism (for **170**), as indicated above. No adverse effects were noted in any of the in vivo experiments and the percentage weight changes for the mice were well within normal thresholds (see the Supporting Information, Table S4).

**Figure 5:**
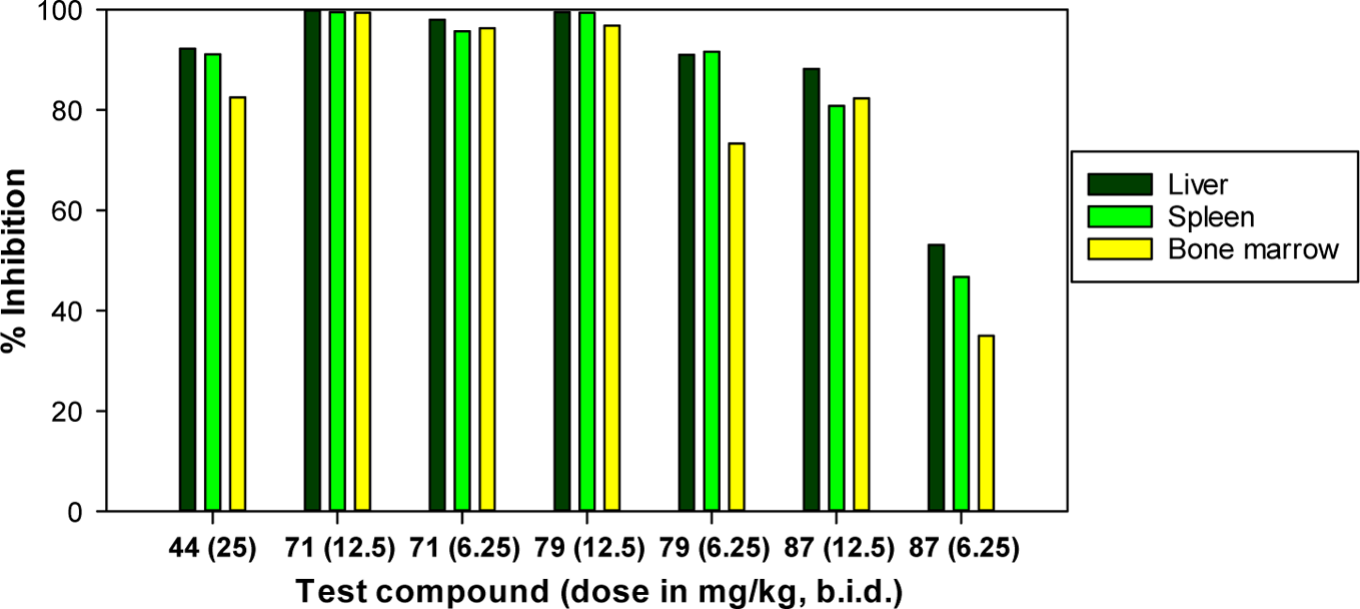
Comparative in vivo efficacy against *L. inf* in the hamster model (LMPH).

Dose–response appraisal of **71** in this mouse model provided an ED_50_ value of 5.1 mg/kg (cf. 4.2 mg/kg for **44**), whereas the trifluoromethoxy analogue **93** was unexpectedly ∼3-fold better (50% at 1.56 mg/kg; Table 3). Therefore, the enantiomers of both **71** and **93** were assessed and in each case the *R* form (**79** and **94**) gave higher efficacy, with **94** (84% at 1.56 mg/kg) outperforming the preclinical candidate **4** (49%). Meanwhile, **71** was further evaluated in the *L. inf* hamster model at LMPH. A dose regimen of 25 or 12.5 mg/kg b.i.d. for 5 days enabled parasite burden reductions exceeding 99% for all three target organs (Table 5 and Figure 5), similar to **4** at 25 mg/kg once daily (qd). However, **71** was slightly less effective than **4** when given via the 12.5 mg/kg qd schedule. A final head-to-head comparison of the enantiomers of **71** confirmed **79** as the preferred stereoisomer based on its superior efficacy at two dose levels. This result was also supported by favorable PK data, e.g., a higher exposure than **87** in hamsters, with an acceptable half-life (3.1 h) and good oral bioavailability (34%) in the rat (Table 4 and Supporting Information, Figure S3). Although **94** was not tested in the hamster model, it is thought that **79** may still offer some advantages as a lead candidate, e.g., lower lipophilicity (by ∼1 log unit) and reduced molecular weight (this could lessen PPB and improve safety),^42^ slightly better solubility (a calculated p*K*_a_ value of 3.76 vs 3.42 for **94**), and a physical form more suitable for oral administration.

In line with our initial objective to develop improved drug candidates as backups to **4**, it was pertinent to examine some additional properties of **79** (Table 6). Compared to **4**, **79** had a very similar molecular weight (370 vs 359 Da) and provided thermodynamic solubility values that were clearly superior to **4** as the pH approached the measured p*K*_a_ value of 3.95. It also had a lower experimental Log *D* value (2.45 vs 3.10), close to that of pretomanid (**6**).^18^ Furthermore, like **4**,^54^ **79** displayed high permeability (without being a substrate for P-gp mediated efflux), although it did show a slightly greater binding to human plasma proteins (96.5 vs 93.9%). In terms of safety, **79** gave a low inhibition of hERG (IC_50_ > 30 *μ*M), did not inhibit CYP3A4 (IC_50_ > 100 *μ*M), and was not mutagenic (Ames test). These characteristics broadly match the suggested criteria for clinical development of a new entity for VL,^55^ so following a belated concern with **4**, **79** has now been selected as a new preclinical candidate.

**Table 6:** Additional Comparative Data for Lead Compounds 4 and 79

property	4	79
molecular weight (Da)	359.3	370.3
Log D (measured)	3.10	2.45^*a*^
p*K*_a_ (measured)		3.95^*a*^
thermodynamic solubility (*μ*M):		
pH 7.4	2.8	5.4^*b*^
pH 6.5/5.0		3.1/18
permeability:		
*P*_app_ (× 10^–6^ cm/s) A to B/B to A	22.6/24.7^*c*^	29.2/26.2^*d*^
human plasma protein binding (%)	93.9	96.5
mutagenic effect (Ames test)	no	no^*a,e*^
hERG IC50 (*μ*M)	10.5	>30
CYP3A4 IC50 (*μ*M)	>25	>100

a For racemate (71).

b Preclinical batch.

c Caco-2 data from ref 54.

d MDCK-MDR1 data; no P-gp mediated efflux.

e Not mutagenic in strains TA98 and TA100, either in the presence or absence of metabolic activation (S9 fraction).

**SAR of 7-Substituted 2-Nitroimidazooxazines for TB**. Although the primary goal of our work with DND*i* was a new drug for VL, the series was originally designed and exemplified for TB, seeking a novel second-generation backup to **6** (now in phase II/III clinical trials^13^). Hence, the antitubercular activities of the 7-substituted oxazine derivatives have remained an aspect of significant ongoing interest. The work began with the preparation of an exploratory set of four compounds (**14**, **22**, **39**, and **45**; Table 1). Growth inhibitory effects against *Mycobacterium tuberculosis* (*M. tb*, strain H37Rv) were studied under both aerobic (replicating) and hypoxic (nonreplicating) conditions (MABA^41^ and LORA^56^ assays, respectively), in recognition of the varying modes of action of **6** under each state^57^ and the suggestion that optimizing for hypoxic activity may lead to agents with better sterilizing ability against persistent bacteria;^56^ recorded MIC data (for at least 90% inhibition) represent the mean of 2–5 independent measurements. Compared with racemic **6** (MICs of 1.1 and 4.4 *μ*M in MABA and LORA, respectively),^14^ compounds **14** and **22** showed potencies of similar magnitude, stimulating further interest and the synthesis of more than 30 new analogues, including **21**, **23**, **25**, **27–29**, **44**, **47**, **49**, and **52–54** (Table 1). These featured two design elements that had proven most advantageous for enhancing in vivo efficacy in early studies of **4** and **6**, namely biaryl extension, and methylation adjacent to the ring oxygen.^11,30,58^

**Figure 6:**
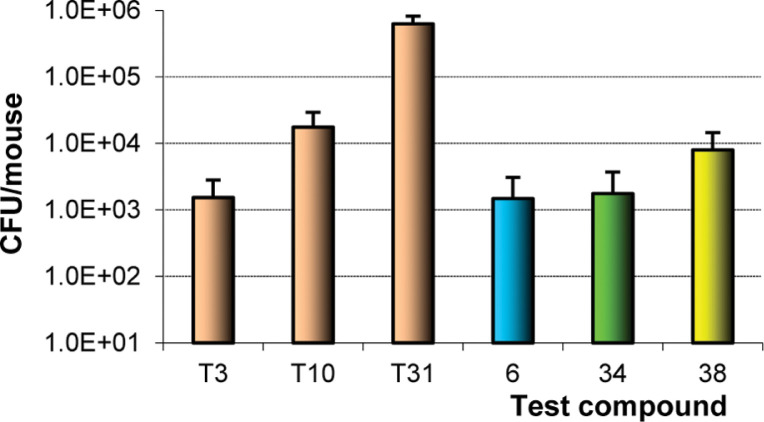
Comparison of **6**, **34**, and **38** in the acute TB infection mouse model.

From this larger data set, it was observed that 7-methyl congeners (e.g., **21**, **23**, **44**, and **47**) were generally slightly more effective than 7-H counterparts and that biphenyl side chains (e.g., **25**, **27–29**, **49**, and **52–54**) provided roughly an order of magnitude further improvement in MABA MIC values (whereas LORA data were less responsive to these changes). The phenylbenzyl derivative **29** was earmarked as a potential early lead based on its better MIC profile (0.093 and 1.4 *μ*M in MABA and LORA) and good stability toward MLM and HLM (77–85% parent remaining after a 1 h exposure, Table 3). Thus, for preliminary proof of principle, the enantiomers of **29** (**34** and **38**) were prepared and assessed in the acute TB infection mouse model alongside **6**, dosing orally at 100 mg/kg daily (5 days/week) for three weeks. In this experiment, the *R* enantiomer **34** displayed equivalent efficacy to **6**, but the *S* form **38** was 5-fold less active (Figure 6), in accordance with its weaker potency and MLM stability data. Nevertheless, the very high lipophilicity of **34** (CLogP 5.52) and its inferior PK profile in comparison to the shorter linked analogue **53** (Table 4) imply that far better in vivo effects might be achievable with optimized compounds (as shown for VL; cf. **94** vs **28**).

The early preference for 7-methyl substitution was not a consistent pattern across heterobiaryl derivatives, where the most potent examples, notably phenylpyridines **59**, **64**, **93** and **94**, as well as phenylpyrimidine **129** (MABA MICs 0.02–0.04 *μ*M), were 7-H compounds. As found for the 6-substituted series,^16^ bipyridine and other heterobiaryl analogues were generally less impressive (except the 4-CF_3_ congener **111**), particularly when the rings were *meta*-linked (corresponding phenylpyridines **99** and **101–103** also displayed markedly reduced activity). Finally, arylated cyclic amine bioisosteres (Table 2) showed moderate to weak potencies overall, with the hydrophilic benzoylpiperazines **157** and **159** being especially poor. For side chains A–C, 7-methyl compounds exhibited an order of magnitude better aerobic activity than their 7-H counterparts, although LORA results were disappointing for these and the related O-linked phenylpiperidines (**170** and **178**). Nevertheless, it was recognized that most of these new 7-substituted oxazines possessed a 4-fluoro substituted terminal ring, whereas more lipophilic 4-trifluoromethoxyphenyl (or 4-CF_3_pyridine) termini were favored for TB^16–18,43,58^ (as seen for phenylpyridines **93–95** vs **71**, **79**, and **87**). Therefore, a few additional examples of the latter type (**189–194** and **198**; Supporting Information, Table S3) were also made and evaluated. Here, **191** and **198** (4-OCF_3_) were 10- to 16-fold more effective than **123** and **139** (4-F) in both TB assays (**192–194** were also 2–8-fold better than **126**, **129**, and **131** in MABA), confirming this same SAR pattern. Overall, taking into account potency^46^ (needing to be superior to **5**^11^), solubility, and metabolism effects, it is considered that phenylpyridine **94** is the most promising lead for TB.

## ■ CONCLUSIONS

Through a scaffold hopping design strategy, 7-substituted 2-nitroimidazooxazines were identified as a third, highly active nitroimidazole-based class of antitubercular agents, having a remarkable similarity in properties to 2-substituted 6-nitroimidazooxazoles. Phenotypic screening of some unoptimized early examples against kinetoplastid diseases led to the detection of two compounds (**44** and **49**) having significant efficacy against VL in mouse and hamster models, although these proved to be inferior to preclinical lead **4** as potential drug candidates. On the basis of our experiences in the original two classes (with **4** and **6**), we then sought to develop more suitable second-generation agents for VL by systematically exploring heterocyclic side chain variants of biphenyl lead **49**. Replacement of one or both phenyl rings by pyridine (or pyrimidine etc.) enabled large modulations in lipophilicity (ΔCLogP –0.5 to –2.7 units), with concomitant improvements in aqueous solubility (2–71-fold at pH 7 and ∼4000-fold at pH 1 for phenyl-3-pyridines). In a complementary bioisostere approach, the incorporation of piperazine or piperidine for the first ring produced even greater solubility enhancements (e.g., **170**: 34 mg/mL at low pH). However, more subtle strategies (viz. *ortho*-substitution of aryl groups and *meta*-linkage of aryl rings) proved less beneficial overall.

Interestingly, potency against *L. inf* appeared to show some dependence on lipophilicity, with the most effective 7-H compounds falling in a CLogP range of 2.9–4.0, and compounds of CLogP < 2.5 having weak or negligible activity. This was aptly demonstrated by the improved potency of fluorinated phenylpyridines (5–16-fold over **49**), in which the pyridine could be either terminal or proximal to the linker, whereas the combination of two pyridine rings was strongly deactivating except in the presence of a 7-methyl substituent. Phenylpyrimidine and phenylpiperidine were the only other side chains to provide substantial activity in this assay. It has recently been shown^59^ that a novel nitroreductase (NTR2) in *Leishmania* is responsible for the activation of nitroimidazooxazoles such as **4**; therefore, differential nitroreductase binding may be a major factor behind the in vitro SARs for both VL and TB.^57^ Evaluation of a representative set of nine racemic compounds in the VL mouse model pinpointed phenylpyridines **71** and **93** as the most efficacious, with **93** being as impressive as **4** (50% inhibition at 1.56 mg/kg). In the chronic infection *L. inf* hamster model, **71** (at 12.5 mg/kg b.i.d.) achieved >99% reductions in parasite burden for all three target organs. Subsequent synthesis and assessment of the enantiomers of both leads identified the *R* forms (**79** and **94**) as superior, and in the case of **79** this outcome was reinforced by excellent results in the *L. inf* hamster model and favorable PK data in the hamster and rat. Importantly, **79** (DNDI-0690) also provided a better safety profile than **4** and has now been selected as a new preclinical candidate for VL.

Finally, as found for the nitroimidazooxazole series,^11^ it was intriguing to note that some of the best VL leads (e.g., **59**, **79**, **93**, **94**, and **129**) showed highly potent in vitro effects against TB, with both *R* enantiomers and 4-trifluoromethoxy analogues most preferred, pointing to **94** (MABA MIC 0.024 *μ*M) as the favored TB candidate for further evaluation. The *S* form of **93** (**95**) also displayed interesting activity against *T. cruzi* (IC_50_ 0.13 *μ*M), indicating a possible application for treating Chagas disease. This investigation has therefore revealed that the 7-substituted 2-nitroimidazooxazine class has exciting potential to treat up to three neglected diseases and can deliver drug candidates that are worthy of examination in ongoing studies.

## ■ EXPERIMENTAL SECTION

Combustion analyses were performed by the Campbell Microanalytical Laboratory, University of Otago, Dunedin, New Zealand. Melting points were determined using an Electrothermal IA9100 melting point apparatus and are as read. NMR spectra were measured on a Bruker Avance 400 spectrometer at 400 MHz for ^1^H and 100 MHz for ^13^C and were referenced to Me_4_Si or solvent resonances. Chemical shifts and coupling constants were recorded in units of ppm and hertz, respectively. High-resolution fast atom bombardment (HRFABMS) mass spectra were determined on a VG-70SE mass spectrometer at nominal 5000 resolution. High-resolution electrospray ionization (HRESIMS) mass spectra were determined on a Bruker micrOTOF-Q II mass spectrometer. Low-resolution atmospheric pressure chemical ionization (APCI) mass spectra were obtained for organic solutions using a ThermoFinnigan Surveyor MSQ mass spectrometer connected to a Gilson autosampler. Optical rotations were measured on a Schmidt + Haensch Polartronic NH8 polarimeter. Column chromatography was performed on silica gel (Merck 230–400 mesh). Thin-layer chromatography was carried out on aluminum-backed silica gel plates (Merck 60 F_254_), with visualization of components by UV light (254 nm), I_2_, or KMnO_4_ staining. Tested compounds (including batches screened in vivo) were ≥95% pure, as determined by combustion analysis (results within 0.4% of theoretical values) and/or by HPLC conducted on an Agilent 1100 system, using a 150 mm × 3.2 mm Altima 5 *μ*m reversed phase C18 column with diode array detection. Preparative reversed phase HPLC was performed using a Gilson Unipoint system (322-H pump, 156 UV/vis detector) with 250 mm × 21 mm Synergi Max-RP 4 *μ*m C12 or Zorbax 7 *μ*m SB-C18 columns. Finally, preparative chiral HPLC was carried out on similar equipment by employing a 250 mm × 20 mm CHIRALPAK IA 5 *μ*m semipreparative column, while chiral purity was assessed using 250 mm × 4.6 mm CHIRALPAK IA or CHIRALPAK AS-H 5 *μ*m analytical columns.

**Compounds of Table 1**. The following section details the syntheses of compounds **14**, **25**, **34**, **44**, **55**, **59**, and **79** of Table 1, via representative procedures and key intermediates, as described in Schemes 1–3. For the syntheses of all of the other compounds in Table 1, please refer to the Supporting Information.

*Synthesis of **14** (Scheme 1A). Procedure A: 2-Bromo-1-(but-3-en-1-yl)-4-nitro-1H-imidazole (**9**)*. A mixture of 2-bromo-4-nitro-1*H*-imidazole (**8**) (2.50 g, 13.0 mmol), 4-bromobut-1-ene (2.00 mL, 19.7 mmol), and powdered K_2_CO_3_ (5.39 g, 39.0 mmol) in anhydrous DMF (25 mL) under N_2_ was stirred at 73 °C for 4.5 h. The resulting cooled mixture was added to ice/aqueous NaHCO_3_ (200 mL) and extracted with EtOAc (4 × 200 mL). The extracts were washed with water (200 mL) and then evaporated to dryness under reduced pressure (at 30 °C), and the residue was chromatographed on silica gel. Elution with 0–10% EtOAc/petroleum ether first gave foreruns, and then further elution with 20% EtOAc/petroleum ether gave **9** (2.96 g, 92%) as a pale-yellow oil that solidified on cooling: mp 28–30 °C. ^1^H NMR (CDCl_3_) *δ* 7.77 (s, 1 H), 5.75 (ddt, *J* = 17.1, 10.2, 6.9 Hz, 1 H), 5.18 (dq, *J* = 10.2, 1.1 Hz, 1 H), 5.12 (dq, *J* = 17.1, 1.4 Hz, 1 H), 4.09 (t, *J* = 7.0 Hz, 2 H), 2.58 (qt, *J* = 6.9, 1.2 Hz, 2 H). HRFABMS calcd for C_7_H_9_BrN_3_O_2_ *m*/*z* [M + H]^+^ 247.9858, 245.9878, found 247.9860, 245.9882.

*Procedure B: 4-(2-Bromo-4-nitro-1H-imidazol-1-yl)butane-1,2-diol (**10**)*. Osmium tetroxide (3.20 mL of a 4% aqueous solution, 0.524 mmol) was added to a solution of alkene **9** (2.56 g, 10.4 mmol) and 4-methylmorpholine 4-oxide (1.83 g, 15.6 mmol) in CH_2_Cl_2_ (65 mL). The mixture was stirred at 20 °C for 4 h, and the resulting precipitate was collected by filtration, washing with CH_2_Cl_2_ and water, to give **10** (2.29 g, 79%) as a cream solid: mp (THF/Et_2_O/pentane) 99–101 °C. ^1^H NMR [(CD_3_)_2_SO] *δ* 8.55 (s, 1 H), 4.77 (br d, *J* = 5.0 Hz, 1 H), 4.58 (br t, *J* = 5.6 Hz, 1 H), 4.20–4.07 (m, 2 H), 3.47–3.37 (m, 1 H), 3.34 (dt, *J* = 10.7, 5.4 Hz, 1 H), 3.24 (dt, *J* = 10.7, 5.9 Hz, 1 H), 2.03–1.92 (m, 1 H), 1.76–1.63 (m, 1 H). Anal. (C_7_H_10_BrN_3_O_4_) C, H, N.

The remaining filtrate above was added to an ice-cold aqueous solution of sodium sulphite (100 mL), and the aqueous portion was saturated with salt and extracted with EtOAc (10 × 100 mL). The combined organic portions were evaporated to dryness under reduced pressure (at 30 °C), and the residue was chromatographed on silica gel. Elution with 0–50% EtOAc/petroleum ether first gave foreruns, and then further elution with EtOAc gave additional **10** (572 mg, 20%).

*Procedure C: 4-(2-Bromo-4-nitro-1H-imidazol-1-yl)-1-[(triisopropylsilyl)oxy]butan-2-ol (**11**)*. Chlorotriisopropylsilane (2.35 mL, 11.0 mmol) was slowly added to a solution of diol **10** (2.86 g, 10.2 mmol) and imidazole (1.54 g, 22.6 mmol) in anhydrous DMF (25 mL) under N_2_, and then the mixture was stirred at 20 °C for 2 d. The resulting mixture was added to ice–water (150 mL) and extracted with 50% EtOAc/petroleum ether (4 × 100 mL). The extracts were washed with water (100 mL) and then concentrated under reduced pressure (at 30 °C), and the remaining oil was chromatographed on silica gel. Elution with 0–20% EtOAc/petroleum ether first gave foreruns, and then further elution with 33% EtOAc/petroleum ether gave **11** (4.19 g, 94%) as a white solid: mp (CH_2_Cl_2_/pentane) 90–91 °C. ^1^H NMR (CDCl_3_) *δ* 7.89 (s, 1 H), 4.24 (dd, *J* = 7.7, 6.2 Hz, 2 H), 3.74 (dd, *J* = 9.6, 3.5 Hz, 1 H), 3.67–3.58 (m, 1 H), 3.53 (dd, *J* = 9.6, 6.8 Hz, 1 H), 2.59 (d, *J* = 3.8 Hz, 1 H), 1.95–1.82 (m, 2 H), 1.18–1.02 (m, 21 H). Anal. (C_16_H_30_BrN_3_O_4_Si) C, H, N.

*Procedure D: 2-Nitro-7-{[(triisopropylsilyl)oxy]methyl}-6,7-dihydro-5H-imidazo[2,1-b][1,3]oxazine (**12**)*. A solution of alcohol **11** (1.89 g, 4.33 mmol) in anhydrous DMF (35 mL) under N_2_ at 0 °C was treated with 60% NaH (262 mg, 6.55 mmol) and then quickly degassed and resealed under N_2_. The mixture was stirred at 0 °C for 25 min and at 20 °C for 3 h, then cooled (CO_2_/acetone), quenched with ice/aqueous NaHCO_3_ (10 mL), added to brine (100 mL), and extracted with CH_2_Cl_2_ (6 × 100 mL). The combined extracts were evaporated to dryness under reduced pressure (at 30 °C), and the residue was chromatographed on silica gel. Elution with 0–20% EtOAc/petroleum ether first gave foreruns, and then further elution with 0–4% EtOAc/CH_2_Cl_2_ gave **12** (1.48 g, 96%) as a pale-yellow solid: mp (CH_2_Cl_2_/pentane) 121–123 °C. ^1^H NMR (CDCl_3_) *δ* 7.42 (s, 1 H), 4.49–4.40 (m, 1 H), 4.17 (ddd, *J* = 12.3, 5.8, 3.7 Hz, 1 H), 4.06 (ddd, *J* = 12.3, 10.3, 5.4 Hz, 1 H), 4.03 (dd, *J* = 10.7, 4.1 Hz, 1 H), 3.95 (dd, *J* = 10.7, 5.8 Hz, 1 H), 2.42–2.33 (m, 1 H), 2.33–2.20 (m, 1 H), 1.18–1.03 (m, 21 H). Anal. (C_16_H_29_N_3_O_4_Si) C, H, N.

*Procedure E: (2-Nitro-6,7-dihydro-5H-imidazo[2,1-b][1,3]oxazin-7-yl)methanol (**13**)*. Silyl ether **12** (1.48 g, 4.16 mmol) was treated with a solution of 1% HCl in 95% EtOH^27^ (63 mL, 15.1 mmol). The mixture was stirred at 20 °C for 36 h and then cooled (CO_2_/acetone) and neutralized with a solution of NH_3_ in MeOH (8.0 mL of 2 M). The resulting mixture was evaporated to dryness under reduced pressure (at 30 °C), and the residue was chromatographed on silica gel. Elution with 0–2% MeOH/CH_2_Cl_2_ first gave foreruns, and then further elution with 2–4% MeOH/CH_2_Cl_2_ gave **13** (804 mg, 97%) as a light-yellow solid: mp (THF/MeOH/CH_2_Cl_2_/hexane) 179–181 °C. ^1^H NMR [(CD_3_)_2_SO] *δ* 8.04 (s, 1 H), 5.12 (t, *J* = 5.8 Hz, 1 H), 4.53–4.43 (m, 1 H), 4.13 (ddd, *J* = 12.5, 5.8, 3.0 Hz, 1 H), 4.04 (ddd, *J* = 12.4, 11.0, 5.1 Hz, 1 H), 3.70–3.59 (m, 2 H), 2.23–2.13 (m, 1 H), 2.10–1.96 (m, 1 H). Anal. (C_7_H_9_N_3_O_4_) C, H, N.

*Procedure F: 2-Nitro-7-({[4-(trifluoromethoxy)benzyl]oxy}-methyl)-6,7-dihydro-5H-imidazo[2,1-b][1,3]oxazine (**14**)*. A solution of alcohol **13** (40.2 mg, 0.202 mmol) in anhydrous DMF (2 mL) under N_2_ at 0 °C was treated with 60% NaH (13.7 mg, 0.343 mmol) and then quickly degassed and resealed under N_2_. 4-(Trifluoromethoxy)benzyl bromide (60 *μ*L, 0.375 mmol) was added, and the mixture was stirred at 20 °C for 165 min, then cooled (CO_2_/acetone), quenched with ice/aqueous NaHCO_3_ (10 mL), added to brine (40 mL), and extracted with CH_2_Cl_2_ (6 × 50 mL). The combined extracts were evaporated to dryness under reduced pressure (at 30 °C), and the residue was chromatographed on silica gel. Elution with 0–0.5% MeOH/CH_2_Cl_2_ first gave foreruns, and then further elution with 0.5% MeOH/CH_2_Cl_2_ gave **14** (52 mg, 69%) as a cream solid: mp (CH_2_Cl_2_/hexane) 158–160 °C. ^1^H NMR [(CD_3_)_2_SO] *δ* 8.06 (s, 1 H), 7.47 (br d, *J* = 8.7 Hz, 2 H), 7.35 (br d, *J* = 7.9 Hz, 2 H), 4.77–4.69 (m, 1 H), 4.60 (s, 2 H), 4.13 (ddd, *J* = 12.5, 5.8, 3.0 Hz, 1 H), 4.05 (ddd, *J* = 12.5, 10.8, 5.2 Hz, 1 H), 3.76 (dd, *J* = 11.1, 3.9 Hz, 1 H), 3.73 (dd, *J* = 11.1, 5.1 Hz, 1 H), 2.27–2.17 (m, 1 H), 2.17–2.03 (m, 1 H). ^13^C NMR [(CD_3_)_2_SO] *δ* 147.9, 147.6 (q, *J_C–F_* = 1.4 Hz), 142.0, 137.6, 129.2 (2 C), 120.9 (2 C), 120.1 (q, *J_C–F_* = 256.0 Hz), 117.7, 76.7, 71.4, 70.9, 41.8, 22.6. Anal. (C_15_H_14_F_3_N_3_O_5_) C, H, N.

*Synthesis of **25** (Scheme 1B). Procedure G: 7-{[(4-Iodobenzyl)-oxy]methyl}-2-nitro-6,7-dihydro-5H-imidazo[2,1-b][1,3]oxazine (**24**)*. A mixture of alcohol **13** (130 mg, 0.653 mmol) and 4-iodobenzyl bromide (262 mg, 0.882 mmol) in anhydrous DMF (5 mL) under N_2_ at 0 °C was treated with 60% NaH (40 mg, 1.00 mmol) and then quickly degassed and resealed under N_2_. The mixture was stirred at 20 °C for 2.5 h, then cooled (CO_2_/acetone), quenched with ice/aqueous NaHCO_3_ (10 mL), added to brine (40 mL), and extracted with CH_2_Cl_2_ (5 × 50 mL). The combined extracts were evaporated to dryness under reduced pressure (at 30 °C), and the residue was chromatographed on silica gel. Elution with CH_2_Cl_2_ first gave foreruns, and then further elution with 1–1.5% EtOAc/CH_2_Cl_2_ gave **24** (165 mg, 61%) as a cream solid: mp (CH_2_Cl_2_/hexane) 169–171 °C. ^1^H NMR (CDCl_3_) *δ* 7.68 (br d, *J* = 8.3 Hz, 2 H), 7.41 (s, 1 H), 7.05 (br d, *J* = 8.2 Hz, 2 H), 4.59–4.52 (m, 3 H), 4.14 (ddd, *J* = 12.3, 5.7, 3.8 Hz, 1 H), 4.05 (ddd, *J* = 12.3, 10.0, 5.6 Hz, 1 H), 3.80 (dd, *J* = 10.6, 4.3 Hz, 1 H), 3.75 (dd, *J* = 10.6, 5.0 Hz, 1 H), 2.37–2.20 (m, 2 H). HRFABMS calcd for C_14_H_15_IN_3_O_4_ *m*/*z* [M + H]^+^ 416.0107, found 416.0105.

*Procedure H: 7-{[(4′-Fluoro[1,1′-biphenyl]-4-yl)methoxy]methyl}-2-nitro-6,7-dihydro-5H-imidazo[2,1-b][1,3]oxazine (**25**)*. A stirred mixture of iodide **24** (35 mg, 0.084 mmol), 4-fluorophenylboronic acid (15.8 mg, 0.113 mmol), and Pd(dppf)Cl_2_ (2.1 mg, 0.003 mmol) in toluene (1.8 mL) and EtOH (0.7 mL) was degassed for 5 min (vacuum pump), and then N_2_ was added. An aqueous solution of Na_2_CO_3_ (0.35 mL of 2 M, 0.70 mmol) was added by syringe, and the mixture was stirred at 90 °C for 20 min, and then cooled, diluted with aqueous NaHCO_3_ (50 mL), and extracted with CH_2_Cl_2_ (4 × 50 mL). The extracts were evaporated to dryness under reduced pressure (at 30 °C), and the residue was chromatographed on silica gel. Elution with 0–1% EtOAc/CH_2_Cl_2_ first gave foreruns, and then further elution with 1–1.5% EtOAc/CH_2_Cl_2_ gave **25** (30.5 mg, 94%) as a cream solid: mp (CH_2_Cl_2_/pentane) 147–149 °C. ^1^H NMR [(CD_3_)_2_SO] *δ* 8.07 (s, 1 H), 7.71 (br dd, *J* = 8.9, 5.4 Hz, 2 H), 7.64 (br d, *J* =8.2 Hz, 2 H), 7.43 (br d, *J* = 8.3 Hz, 2 H), 7.29 (br t, *J* = 8.9 Hz, 2 H), 4.77–4.69 (m, 1 H), 4.61 (s, 2 H), 4.13 (ddd, *J* = 12.5, 5.8, 3.0 Hz, 1 H), 4.05 (ddd, *J* = 12.4, 10.9, 5.2 Hz, 1 H), 3.77 (dd, *J* = 11.0, 3.9 Hz, 1 H), 3.74 (dd, *J* = 11.1, 5.1 Hz, 1 H), 2.28–2.17 (m, 1 H), 2.17–2.03 (m, 1 H). ^13^C NMR [(CD_3_)_2_SO] *δ* 161.9 (d, *J_C–F_* = 244.4 Hz), 148.0, 142.0, 138.4, 137.2, 136.3 (d, *J_C–F_* = 3.0 Hz), 128.6 (d, *J_C–F_* = 8.1 Hz, 2 C), 128.1 (2 C), 126.6 (2 C), 117.7, 115.7 (d, *J_C–F_* = 21.2 Hz, 2 C), 76.8, 72.0, 70.8, 41.8, 22.6. Anal. (C_20_H_18_FN_3_O_4_) C, H, N.

*Synthesis of **34** (Scheme 1C). (7-Methyl-2-nitro-6,7-dihydro-5H-imidazo[2,1-b][1,3]oxazin-7-yl)methyl acetate (**30**)*. Acetic anhydride (3.60 mL, 38.1 mmol) was added to a suspension of alcohol **20** (see Supporting Information) (807 mg, 3.79 mmol) in anhydrous pyridine (7.0 mL). The mixture was stirred at 20 °C for 38 h and then added to ice–water (150 mL) and extracted with CH_2_Cl_2_ (5 × 100 mL). The extracts were evaporated to dryness under reduced pressure (at 30 °C), and the residue was chromatographed on silica gel. Elution with CH_2_Cl_2_ first gave foreruns, and then further elution with 1–6% EtOAc/CH_2_Cl_2_ gave **30** (962 mg, 100%) as a cream solid: mp (CH_2_Cl_2_/pentane) 145–147 °C. ^1^H NMR (CDCl_3_) *δ* 7.44 (s, 1 H), 4.27 (d, *J* = 11.9 Hz, 1 H), 4.20 (d, *J* = 11.9 Hz, 1 H), 4.14 (dt, *J* = 12.7, 5.9 Hz, 1 H), 4.08 (ddd, *J* = 12.7, 8.3, 5.6 Hz, 1 H), 2.32 (ddd, *J* = 14.5, 8.3, 6.1 Hz, 1 H), 2.10 (dt, *J* = 14.5, 5.7 Hz, 1 H), 2.09 (s, 3 H), 1.50 (s, 3 H). HRFABMS calcd for C_10_H_14_N_3_O_5_ *m*/*z* [M + H]^+^ 256.0934, found 256.0941.

*[(7R)-7-Methyl-2-nitro-6,7-dihydro-5H-imidazo[2,1-b][1,3]-oxazin-7-yl]methyl acetate (**31**) and [(7S)-7-methyl-2-nitro-6,7-dihydro-5H-imidazo[2,1-b][1,3]oxazin-7-yl]methyl acetate (**35**)*. Racemic acetate **30** (990 mg) was separated into pure enantiomers by preparative chiral HPLC, using a CHIRALPAK IA column and an isocratic solvent system of 40% EtOH in hexane at a flow rate of 6 mL/min, to first give **35** (427 mg, 43%) as a cream solid, having identical ^1^H NMR data to **30** that was used directly in the next step; [*α*]^26^_D_ –6.0 (*c* 1.00, CHCl_3_).

Further elution of the HPLC column gave **31** (428 mg, 43%) as a cream solid that was used directly in the next step. ^1^H NMR (CDCl_3_) *δ* 7.44 (s, 1 H), 4.27 (d, *J* = 11.9 Hz, 1 H), 4.20 (d, *J* = 11.8 Hz, 1 H), 4.14 (dt, *J* = 12.7, 5.9 Hz, 1 H), 4.08 (ddd, *J* = 12.7, 8.3, 5.6 Hz, 1 H), 2.32 (ddd, *J* = 14.5, 8.3, 6.1 Hz, 1 H), 2.10 (dt, *J* = 14.5, 5.7 Hz, 1 H), 2.09 (s, 3 H), 1.50 (s, 3 H). [*α*]^26^_D_ 6.0 (*c* 1.00, CHCl_3_). Chiral HPLC (using a CHIRALPAK IA analytical column and eluting with 40% EtOH in hexane at 0.5 mL/min) determined that the ee of each enantiomer was 100%.

*Procedure I: [(7R)-7-Methyl-2-nitro-6,7-dihydro-5H-imidazo[2,1-b][1,3]oxazin-7-yl]methanol (**32**)*. A stirred solution of ester **31** (427 mg, 1.67 mmol) in MeOH (36 mL) was treated with K_2_CO_3_ (256 mg, 1.85 mmol), and then water (4 mL) was added dropwise. The mixture was stirred at 20 °C for 4 h and then cooled in ice and neutralized with 0.1 M HCl (37 mL). The resulting mixture was evaporated to dryness under reduced pressure (at 30 °C), and the residue was chromatographed on silica gel. Elution with 0–1% MeOH/CH_2_Cl_2_ first gave foreruns, and then further elution with 1–2.5% MeOH/CH_2_Cl_2_ gave **32** (343 mg, 96%) as a light-yellow solid that was used directly in the next step. ^1^H NMR [(CD_3_)_2_SO] *δ* 8.03 (s, 1 H), 5.23 (br t, *J* =5.4Hz, 1 H), 4.13 (dt, *J* = 13.0, 6.0 Hz, 1 H), 4.05 (ddd, *J* = 12.9, 8.1, 5.6 Hz, 1 H), 3.54 (dd, *J* = 11.6, 4.9 Hz, 1 H), 3.48 (dd, *J* = 11.6, 5.2 Hz, 1 H), 2.21 (ddd, *J* = 14.4, 8.1, 5.9 Hz, 1 H), 2.00 (dt, *J* = 14.4, 5.8 Hz, 1 H), 1.32 (s, 3 H). [*α*]^27^_D_ –18.0 (*c* 1.00, DMF).

*(7R)-7-{[(4-Bromobenzyl)oxy]methyl}-7-methyl-2-nitro-6,7-dihydro-5H-imidazo[2,1-b][1,3]oxazine (**33**)*. Reaction of alcohol **32** with 4-bromobenzyl bromide (1.3 equiv) and NaH, using procedure G for 3 h, followed by chromatography of the product on silica gel, eluting with CH_2_Cl_2_ (foreruns) and then with 1% EtOAc/CH_2_Cl_2_, gave **33** (57%) as a white solid: mp (CH_2_Cl_2_/hexane) 157–159 °C. ^1^H NMR (CDCl_3_) *δ* 7.46 (br d, *J* = 8.3 Hz, 2 H), 7.39 (s, 1 H), 7.12 (br d, *J* = 8.3 Hz, 2 H), 4.50 (s, 2 H), 4.09 (ddd, *J* = 12.5, 6.9, 6.0 Hz, 1 H), 4.01 (ddd, *J* = 12.5, 7.0, 6.0 Hz, 1 H), 3.62 (d, *J* = 10.2 Hz, 1 H), 3.58 (d, *J* = 10.2 Hz, 1 H), 2.37 (ddd, *J* = 14.4, 7.0, 6.0 Hz, 1 H), 2.10 (ddd, *J* = 14.4, 6.9, 6.1 Hz, 1 H), 1.46 (s, 3 H). [*α*]^27^_D_ 31.0 (*c* 1.00, CHCl_3_). HRFABMS calcd for C_15_H_17_BrN_3_O_4_ *m*/*z* [M + H]^+^ 384.0382, 382.0402, found 384.0385, 382.0398.

*(7R)-7-Methyl-2-nitro-7-({[4′-(trifluoromethoxy)[1,1′-biphenyl]-4-yl]methoxy}methyl)-6,7-dihydro-5H-imidazo[2,1-b][1,3]oxazine(**34**)*. Reaction of bromide **33** with 4-(trifluoromethoxy)phenylboronic acid (1.5 equiv) and Pd(dppf)Cl_2_ (0.15 equiv), using procedure H at 88 °C for 75 min, followed by chromatography of the product on silica gel, eluting with 0–0.5% EtOAc/CH_2_Cl_2_ (foreruns) and then with 0.5–1.5% EtOAc/CH_2_Cl_2_, gave **34** (90%) as a cream solid: mp (CH_2_Cl_2_/hexane) 165–167 °C. ^1^H NMR (CDCl_3_) *δ* 7.58 (br d, *J* = 8.7 Hz, 2 H), 7.52 (br d, *J* = 8.2 Hz, 2 H), 7.38 (s, 1 H), 7.32 (br d, *J* = 8.1 Hz, 2 H), 7.28 (br d, *J* = 8.1 Hz, 2 H), 4.61 (d, *J* = 12.1 Hz, 1 H), 4.58 (d, *J* = 12.1 Hz, 1 H), 4.11 (ddd, *J* = 12.4, 7.2, 5.8 Hz, 1 H), 4.01 (ddd, *J* = 12.6, 6.5, 6.1 Hz, 1 H), 3.67 (d, *J* = 10.2 Hz, 1 H), 3.63 (d, *J* = 10.2 Hz, 1 H), 2.40 (ddd, *J* = 14.4, 6.6, 6.1 Hz, 1 H), 2.13 (ddd, *J* = 14.5, 7.3, 6.0 Hz, 1 H), 1.48 (s, 3 H). [*α*]^27^_D_ 37.0 (*c* 1.00, CHCl_3_). Anal. (C_22_H_20_F_3_N_3_O_5_) C, H, N.

*Synthesis of **44** (Scheme 2B). 2-Chloro-1-(3-methylbut-3-en-1-yl)-4-nitro-1H-imidazole (**41**)*. Reaction of 2-chloro-4-nitro-1*H*-imidazole (**40**) with 4-iodo-2-methylbut-1-ene^26^ (**15**) (1.1 equiv) and powdered K_2_CO_3_ (2.0 equiv), using procedure A for 14 h, gave **41** (84%) as a white solid: mp (CH_2_Cl_2_/petroleum ether) 70–72 °C. ^1^H NMR (CDCl_3_) *δ* 7.72 (s, 1 H), 4.93–4.87 (m, 1 H), 4.72–4.66 (m, 1 H), 4.13 (t, *J* = 7.1 Hz, 2 H), 2.52 (br t, *J* = 7.0 Hz, 2 H), 1.80 (br s, 3 H). Anal. (C_8_H_10_ClN_3_O_2_) C, H, N.

*2-Chloro-1-[2-(2-methyloxiran-2-yl)ethyl]-4-nitro-1H-imidazole(**42**)*. 3-Chloroperoxybenzoic acid (14.4 g of 70%, 58.4 mmol) was added to a mixture of alkene **41** (10.4 g, 48.2 mmol) and disodium hydrogen phosphate (10.4 g, 73.3 mmol) in CH_2_Cl_2_ (300 mL) at 0 °C. The mixture was stirred at 20 °C for 4 h, and then additional *m*-CPBA (2.40 g, 9.74 mmol) and CH_2_Cl_2_ (50 mL) were added. The resulting mixture was stirred at 20 °C for a further 14 h and then cooled to –20 °C and washed with an ice-cold aqueous solution of sodium sulphite (200 mL of 10%), back-extracting with CH_2_Cl_2_ (2 × 200 mL). The organic portions were sequentially washed with aqueous NaHCO_3_ (200 mL) and brine (100 mL) and then combined and concentrated under reduced pressure, and the remaining oil was chromatographed on silica gel. Elution with 3:1 CH_2_Cl_2_/petroleum ether first gave foreruns, and then further elution with 3:1 CH_2_Cl_2_/petroleum ether and 0–2.5% EtOAc/CH_2_Cl_2_ gave **42** (10.6 g, 95%) as a cream solid: mp (CH_2_Cl_2_/petroleum ether) 87–89 °C. ^1^H NMR (CDCl_3_) *δ* 7.79 (s, 1 H), 4.13 (t, *J* = 7.6 Hz, 2 H), 2.67 (br d, *J* = 4.4 Hz, 1 H), 2.62 (br d, *J* = 4.3 Hz, 1 H), 2.19 (dt, *J* = 14.3, 7.7 Hz, 1 H), 2.04 (dt, *J* = 14.3, 7.4 Hz, 1 H), 1.40 (s, 3 H). Anal. (C_8_H_10_ClN_3_O_3_)C, H, N.

*Procedure J: 4-(2-Chloro-4-nitro-1H-imidazol-1-yl)-2-methyl-1-[4-(trifluoromethoxy)phenoxy]butan-2-ol (**43**)*. 4-(Trifluoromethoxy)phenol (0.280 mL, 2.16 mmol) was added to a mixture of epoxide **42** (200 mg, 0.863 mmol) and powdered K_2_CO_3_ (422 mg, 3.05 mmol) in anhydrous MEK (2.0 mL) under N_2_, and then the mixture was stirred at 82 °C for 10 h. The resulting cooled mixture was diluted with water (50 mL) and extracted with CH_2_Cl_2_ (4 × 50 mL). The combined extracts were evaporated to dryness under reduced pressure (at 30 °C), and the residue was chromatographed on silica gel. Elution with CH_2_Cl_2_ first gave foreruns, and then further elution with 0–2% EtOAc/CH_2_Cl_2_ gave **43** (272 mg, 77%) as a pale-yellow oil. ^1^H NMR (CDCl_3_) *δ* 7.81 (s, 1 H), 7.17 (br d, *J* =9.1 Hz,2 H), 6.90 (br d, *J* = 9.2 Hz, 2 H), 4.33–4.20 (m, 2 H), 3.85 (d, *J* = 9.0 Hz, 1 H), 3.82 (d, *J* = 9.0 Hz, 1 H), 2.23 (ddd, *J* = 13.8, 9.3, 6.5 Hz, 1 H), 2.21 (s, 1 H), 2.04 (ddd, *J* = 13.8, 9.6, 6.6 Hz, 1 H), 1.40 (s, 3 H). HRESIMS calcd for C_15_H_16_ClF_3_N_3_O_5_ *m*/*z* [M + H]^+^ 412.0697, 410.0725, found 412.0700, 410.0722.

*7-Methyl-2-nitro-7-{[4-(trifluoromethoxy)phenoxy]methyl}-6,7-dihydro-5H-imidazo[2,1-b][1,3]oxazine (**44**)*. Reaction of alcohol **43** with NaH (1.7 equiv), using procedure D for 2 h, followed by chromatography of the product on silica gel, eluting with CH_2_Cl_2_, gave **44** (61%) as a cream solid: mp (CH_2_Cl_2_/pentane) 134–136 °C. ^1^H NMR [(CD_3_)_2_SO] *δ* 8.10 (s, 1 H), 7.31 (br d, *J* = 9.0 Hz, 2 H), 7.07 (br d, *J* = 9.2 Hz, 2 H), 4.20 (s, 2 H), 4.19 (dt, *J* = 13.3, 6.1 Hz, 1 H), 4.13 (ddd, *J* = 13.2, 8.1, 5.6 Hz, 1 H), 2.38 (ddd, *J* = 14.4, 7.9, 6.2 Hz, 1 H), 2.18 (dt, *J* = 14.4, 5.8 Hz, 1 H), 1.49 (s, 3 H). ^13^C NMR [(CD_3_)_2_SO] *δ* 157.0, 147.2, 142.2, 142.1 (q, *J_C–F_* = 1.6 Hz), 122.5 (2 C), 120.1 (q, *J_C–F_* = 255.2 Hz), 117.7, 115.9 (2 C), 80.4, 72.4, 39.5, 27.0, 21.3. Anal. (C_15_H_14_F_3_N_3_O_5_) C, H, N.

*Synthesis of **55** (Scheme 2A). Procedure K: 7-[(4-Iodophenoxy)-methyl]-2-nitro-6,7-dihydro-5H-imidazo[2,1-b][1,3]oxazine (**48**)*. DEAD (0.270 mL, 1.74 mmol) was added dropwise to a stirred solution of alcohol **13** (251 mg, 1.26 mmol), 4-iodophenol (377 mg, 1.71 mmol), and PPh_3_ (448 mg, 1.71 mmol) in anhydrous THF (3 mL) under N_2_ at 0 °C. After being stirred at 20 °C for 32 h, the mixture was concentrated under reduced pressure to give an oil, which was chromatographed on silica gel. Elution with CH_2_Cl_2_ first gave foreruns, and then further elution with 0–2% EtOAc/CH_2_Cl_2_ gave the crude product, which was further purified by chromatography on silica gel. Elution with 33–50% EtOAc/petroleum ether first gave foreruns, and then further elution with 10% MeOH/CH_2_Cl_2_ gave **48** (433 mg, 86%) as a cream solid: mp (MeOH/CH_2_Cl_2_/hexane) 224–227 °C. ^1^H NMR [(CD_3_)_2_SO] *δ* 8.08 (s, 1 H), 7.62 (br d, *J* = 9.0 Hz, 2 H), 6.86 (br d, *J* = 9.0 Hz, 2 H), 4.94–4.85 (m, 1 H), 4.31 (dd, *J* = 11.1, 3.4 Hz, 1 H), 4.25 (dd, *J* = 11.1, 5.8 Hz, 1 H), 4.18 (ddd, *J* = 12.6, 5.8, 3.0 Hz, 1 H), 4.09 (ddd, *J* = 12.5, 10.8, 5.2 Hz, 1 H), 2.35–2.26 (m, 1 H), 2.25–2.12 (m, 1 H). Anal. (C_13_H_12_IN_3_O_4_) C, H, N.

*Procedure L: 7-{[4-(6-Fluoropyridin-3-yl)phenoxy]methyl}-2-nitro-6,7-dihydro-5H-imidazo[2,1-b][1,3]oxazine (**55**)*. A stirred mixture of iodide **48** (70.3 mg, 0.175 mmol), (6-fluoropyridin-3-yl)boronic acid (42.3 mg, 0.300 mmol), and Pd(dppf)Cl_2_ (19.5 mg, 0.0266 mmol) in DMF (2.3 mL), toluene (1.5 mL), and EtOH (1.0 mL) was degassed for 8 min (vacuum pump), and then N_2_ was added. An aqueous solution of KHCO_3_ (0.40 mL of 2 M, 0.80 mmol) was added by syringe, and the stirred mixture was again degassed for 9 min and then N_2_ was added. The resulting mixture was stirred at 85 °Cfor 2 h, and then cooled, diluted with aqueous NaHCO_3_ (50 mL), and extracted with CH_2_Cl_2_ (6 × 50 mL). The extracts were evaporated to dryness under reduced pressure (at 30 °C), and the residue was chromatographed on silica gel. Elution with 0–4% EtOAc/CH_2_Cl_2_ first gave foreruns, and then further elution with 4–7% EtOAc/CH_2_Cl_2_ gave **55** (61 mg, 94%) as a cream solid: mp (MeOH/CH_2_Cl_2_/hexane) 197–198 °C. ^1^H NMR [(CD_3_)_2_SO] *δ* 8.51 (br d, *J* = 2.6 Hz, 1 H), 8.24 (td, *J* = 8.2, 2.6 Hz, 1 H), 8.10 (s, 1 H), 7.69 (br d, *J* = 8.8 Hz, 2 H), 7.24 (dd, *J* = 8.6, 2.6 Hz, 1 H), 7.13 (br d, *J* = 8.8 Hz, 2 H), 4.99–4.88 (m, 1 H), 4.39 (dd, *J* = 11.1, 3.3 Hz, 1 H), 4.33 (dd, *J* = 11.1, 5.8 Hz, 1 H), 4.20 (ddd, *J* = 12.5, 5.7, 2.9 Hz, 1 H), 4.11 (ddd, *J* = 12.4, 10.9, 5.2 Hz, 1 H), 2.39–2.29 (m, 1 H), 2.29–2.15 (m, 1 H). ^13^C NMR [(CD_3_)_2_SO] *δ* 162.2 (d, *J_C–F_* = 235.1 Hz), 158.2, 147.8, 144.8 (d, *J_C–F_* = 15.1 Hz), 142.1, 139.8 (d, *J_C–F_* = 8.0 Hz), 133.7 (d, *J_C–F_* = 4.6 Hz), 128.7, 128.1 (2 C), 117.8, 115.3 (2 C), 109.5 (d, *J_C–F_* = 37.7 Hz), 76.0, 68.8, 41.7, 22.4. Anal. (C_18_H_15_FN_4_O_4_) C, H, N.

*Synthesis of **59** (Scheme 2C). Procedure M: 7-{[(5-Bromopyridin-2-yl)oxy]methyl}-2-nitro-6,7-dihydro-5H-imidazo[2,1-b][1,3]oxazine (**58**)*. A mixture of alcohol **13** (500 mg, 2.51 mmol) and 5-bromo-2-fluoropyridine (**57**) (0.52 mL, 5.05 mmol) in anhydrous DMF (10 mL) under N_2_ at 0 °C was treated with 60% NaH (151 mg, 3.78 mmol) and then quickly degassed and resealed under N_2_. Further **57** (0.52 mL, 5.05 mmol) was added, and the mixture was stirred at 20 °C for 2.5 h. The resulting mixture was cooled (CO_2_/acetone), quenched with ice/aqueous NaHCO_3_ (20 mL), and then added to brine (100 mL) and extracted with CH_2_Cl_2_ (8 × 100 mL). The combined extracts were evaporated to dryness under reduced pressure (at 30 °C), and the residue was chromatographed on silica gel. Elution with 0–1% EtOAc/CH_2_Cl_2_ first gave foreruns, and then further elution with 2–4% EtOAc/CH_2_Cl_2_ gave **58** (778 mg, 87%) as a white solid: mp (MeOH/CH_2_Cl_2_/hexane) 182–184 °C. ^1^H NMR [(CD_3_)_2_SO] *δ* 8.30 (br d, *J* = 2.6 Hz, 1 H), 8.07 (s, 1 H), 7.94 (dd, *J* = 8.8, 2.6 Hz, 1 H), 6.91 (br d, *J* = 8.8 Hz, 1 H), 4.95–4.86 (m, 1 H), 4.58 (dd, *J* = 12.0, 3.3 Hz, 1 H), 4.52 (dd, *J* = 12.0, 6.0 Hz, 1 H), 4.17 (ddd, *J* = 12.6, 5.8, 2.8 Hz, 1 H), 4.09 (ddd, *J* = 12.5, 11.0, 5.2 Hz, 1 H), 2.35–2.25 (m, 1 H), 2.24–2.10 (m, 1 H). Anal. (C_12_H_11_BrN_4_O_4_) C, H, N.

*Procedure N: 7-({[5-(4-Fluorophenyl)pyridin-2-yl]oxy}methyl)-2-nitro-6,7-dihydro-5H-imidazo[2,1-b][1,3]oxazine (**59**)*. A stirred mixture of bromide **58** (150 mg, 0.422 mmol), 4-fluorophenylboronic acid (117 mg, 0.836 mmol), and Pd(dppf)Cl_2_ (83.1 mg, 0.114 mmol) in DMF (4.5 mL), toluene (3 mL), and EtOH (2 mL) was degassed for 10 min (vacuum pump), and then N_2_ was added. An aqueous solution of Na_2_CO_3_ (1.05 mL of 2 M, 2.1 mmol) was added by syringe, the stirred mixture was again degassed for 10 min, and then N_2_ was added. The resulting mixture was stirred at 89 °C for 2.5 h and then cooled, diluted with aqueous NaHCO_3_ (50 mL), and extracted with CH_2_Cl_2_ (6 × 50 mL). The combined extracts were evaporated to dryness under reduced pressure (at 30 °C), and the residue was chromatographed on silica gel. Elution with 0–3% EtOAc/CH_2_Cl_2_ first gave foreruns, and then further elution with 3% EtOAc/CH_2_Cl_2_ gave **59** (143 mg, 91%) as a cream solid: mp (MeOH/CH_2_Cl_2_/hexane) 180–181 °C. ^1^H NMR [(CD_3_)_2_SO] *δ* 8.47 (br d, *J* = 2.6 Hz, 1 H), 8.09 (s, 1 H), 8.05 (dd, *J* = 8.6, 2.6 Hz, 1 H), 7.71 (br dd, *J* =8.9, 5.4 Hz, 2 H), 7.30 (br t, *J* = 8.9 Hz, 2 H), 6.98 (br d, *J* = 8.6 Hz, 1 H), 4.99–4.90 (m, 1 H), 4.64 (dd, *J* = 12.0, 3.4 Hz, 1 H), 4.58 (dd, *J* = 12.0, 6.0 Hz, 1 H), 4.19 (ddd, *J* = 12.5, 5.8, 2.7 Hz, 1 H), 4.10 (ddd, *J* = 12.5, 11.1, 5.1 Hz, 1 H), 2.38–2.28 (m, 1 H), 2.27–2.13 (m, 1 H). ^13^C NMR [(CD_3_)_2_SO] *δ* 162.1, 161.9 (d, *J_C–F_* = 244.4 Hz), 147.8, 144.5, 142.0, 137.9, 133.3 (d, *J_C–F_* = 3.1 Hz), 128.9, 128.4 (d, *J_C–F_* = 8.1 Hz, 2 C), 117.8, 115.8 (d, *J_C–F_* = 21.5 Hz, 2 C), 110.8, 76.0, 66.3, 41.7, 22.5. Anal. (C_18_H_15_FN_4_O_4_) C, H, N.

*Synthesis of **79** (Scheme 3B). 2-Chloro-1-{2-[(4R)-2,2-dimethyl-1,3-dioxolan-4-yl]ethyl}-4-nitro-1H-imidazole (**73**)*. Reaction of 2-chloro-4-nitro-1*H*-imidazole (**40**) with (4*R*)-4-(2-iodoethyl)-2,2-dimethyl-1,3-dioxolane^33^ (**72**) (0.96 equiv) and powdered K_2_CO_3_ (1.03 equiv), using procedure A for 3 d, followed by chromatography of the product on silica gel, eluting with 0–33% Et_2_O/petroleum ether (foreruns) and then with 33–50% Et_2_O/petroleum ether, gave **73** (74%) as a light-yellow solid: mp (Et_2_O/pentane) 73–75 °C. ^1^H NMR (CDCl_3_) *δ* 7.81 (s, 1 H), 4.23 (ddd, *J* = 14.2, 7.7, 5.3 Hz, 1 H), 4.18 (ddd, *J* = 14.2, 8.0, 7.1 Hz, 1 H), 4.10 (dd, *J* = 7.9, 6.1 Hz, 1 H), 4.09–4.01 (m, 1 H), 3.60 (dd, *J* = 7.8, 5.7 Hz, 1 H), 2.12–2.01 (m, 1 H), 2.01–1.90 (m, 1 H), 1.43 (s, 3 H), 1.36 (s, 3 H). [*α*]^26^_D_ 39.2 (*c* 1.020, CHCl_3_). Anal. (C_10_H_14_ClN_3_O_4_) C, H, N.

*Procedure O: (2R)-4-(2-Chloro-4-nitro-1H-imidazol-1-yl)butane-1,2-diol (**74**)*. Dilute HCl (13 mL of a 1 M solution, 13.0 mmol) was added dropwise to a stirred solution of acetonide **73** (2.86 g, 10.4 mmol) in MeOH (39 mL) at 0 °C. The mixture was stirred at 20 °C for 6 h and then cooled in ice, treated with K_2_CO_3_ (0.90 g, 6.51 mmol), and stirred until the neutralization was complete. Following filtration to remove inorganic material (washing with MeOH), the solvents were removed under reduced pressure (at 30 °C), and the residue was chromatographed on silica gel. Elution with 0–67% EtOAc/petroleum ether first gave foreruns, and then further elution with EtOAc gave **74** (2.39 g, 98%) as a cream solid: mp (MeOH/CH_2_Cl_2_/hexane) 115–117 °C. ^1^H NMR [(CD_3_)_2_SO] *δ* 8.55 (s, 1 H), 4.79 (d, *J* = 5.0 Hz, 1 H), 4.60 (t, *J* = 5.6 Hz, 1 H), 4.21–4.08 (m, 2 H), 3.45–3.36 (m, 1 H), 3.36–3.29 (m, 1 H), 3.22 (dt, *J* = 10.7, 5.9 Hz, 1 H), 2.03–1.91 (m, 1 H), 1.75–1.62 (m, 1 H). [*α*]^24^_D_ 29.4 (*c* 2.008, DMF). Anal. (C_7_H_10_ClN_3_O_4_) C, H, N.

*Procedure P: (2R)-4-(2-Chloro-4-nitro-1H-imidazol-1-yl)-2-hydroxybutyl 4-methylbenzenesulfonate (**75**)*. A solution of tosyl chloride (2.28 g, 12.0 mmol) in anhydrous pyridine (3 mL, then 2 × 1.5 mL to rinse) was added dropwise to a stirred solution of diol **74** (2.35 g, 9.97 mmol) in anhydrous pyridine (5 mL) under N_2_ at –10 °C. The mixture was stirred at –10 to 0 °C for 2 h and then at 20 °C for 13 h. The resulting solution was cooled in ice and then added to ice–water (100 mL) and extracted with CH_2_Cl_2_ (4 × 100 mL). The combined extracts were concentrated to dryness under reduced pressure (at 30 °C), and the remaining oil was chromatographed on silica gel. Elution with 0–2% EtOAc/CH_2_Cl_2_ first gave foreruns, and then further elution with 2–50% EtOAc/CH_2_Cl_2_ gave **75** (3.38 g, 87%) as a cream foam that was used directly in the next step. ^1^HNMR (CDCl_3_) *δ* 7.78 (s, 1 H), 7.78 (br d, *J* = 8.3 Hz, 2 H), 7.37 (br d, *J* = 8.0 Hz, 2 H), 4.22 (dd, *J* = 7.7, 6.0 Hz, 2 H), 4.04 (dd, *J* = 10.5, 3.4 Hz, 1 H), 3.95 (dd, *J* = 10.5, 6.6 Hz, 1 H), 3.87–3.78 (m, 1 H), 2.62 (br d, *J* = 4.3 Hz, 1 H), 2.47 (s, 3 H), 1.99–1.83 (m, 2 H). APCI MS *m*/*z* 392, 390 [M + H]^+^.

*Procedure Q: 2-Chloro-4-nitro-1-{2-[(2R)-oxiran-2-yl]ethyl}-1H-imidazole (**76**)*. 1,8-Diazabicyclo[5.4.0]undec-7-ene (1.45 mL, 9.70 mmol) was added dropwise to a stirred solution of tosylate **75** (3.38 g, 8.67 mmol) in anhydrous CH_2_Cl_2_ (32 mL) under N_2_ at 0 °C. The mixture was stirred at 0 °C for 3h, at 0–20 °C for 2 h, and then at 20 °C for 3 h. The resulting solution was added to a mixture of ice and brine (100 mL) and extracted with CH_2_Cl_2_ (4 × 100 mL). The combined extracts were concentrated to dryness under reduced pressure (at 30 °C), and the remaining oil was chromatographed on silica gel. Elution with CH_2_Cl_2_ first gave foreruns, and then further elution with CH_2_Cl_2_ gave **76** (1.78 g, 94%) as a cream solid (after freezing): mp (CH_2_Cl_2_/pentane) 59–61 °C. ^1^H NMR (CDCl_3_) *δ* 7.81 (s, 1 H), 4.29–4.15 (m, 2 H), 2.99–2.91 (m, 1 H), 2.86 (dd, *J* = 4.7, 4.0 Hz, 1 H), 2.54 (dd, *J* = 4.8, 2.6 Hz, 1 H), 2.36–2.25 (m, 1 H), 1.87–1.76 (m, 1 H). [*α*]^25^_D_ 43.6 (*c* 1.009, CHCl_3_). Anal. (C_7_H_8_ClN_3_O_3_) C, H, N.

*Procedure R: (2R)-1-[(6-Bromopyridin-3-yl)oxy]-4-(2-chloro-4-nitro-1H-imidazol-1-yl)butan-2-ol (**77**)*. A mixture of epoxide **76** (1.76 g, 8.07 mmol), 6-bromopyridin-3-ol (**68**) (2.82 g, 16.2 mmol), and powdered K_2_CO_3_ (2.23 g, 16.1 mmol) in anhydrous MEK (21 mL) under N_2_ was stirred at 80–82 °C for 42 h. The resulting cooled mixture was added to water (100 mL), washing in residues with MeOH/CH_2_Cl_2_, and then extracted with 10% MeOH/CH_2_Cl_2_ (3 × 100 mL) and 25% EtOAc/CH_2_Cl_2_ (3 × 100 mL). The combined extracts were concentrated to dryness under reduced pressure, and the remaining oil was chromatographed on silica gel. Elution with 0–40% EtOAc/petroleum ether first gave foreruns, and then further elution with 50% EtOAc/petroleum ether gave **77** (1.71 g, 54%) as a cream solid: mp (MeOH/CH_2_Cl_2_/hexane) 134–135 °C. ^1^H NMR [(CD_3_)_2_SO] *δ* 8.58 (s, 1 H), 8.12 (d, *J* = 3.1 Hz, 1 H), 7.54 (d, *J* = 8.7 Hz, 1 H), 7.39 (dd, *J* = 8.8, 3.2 Hz, 1 H), 5.30 (d, *J* = 4.9 Hz, 1 H), 4.27–4.14 (m, 2 H), 3.99 (dd, *J* = 10.0, 4.8 Hz, 1 H), 3.95 (dd, *J* = 10.0, 5.5 Hz, 1 H), 3.86–3.77 (m, 1 H), 2.11–2.00 (m, 1 H), 1.95–1.83 (m, 1 H). [*α*]^24^_D_ 7.95 (*c* 1.006, DMF). Anal. (C_12_H_12_BrClN_4_O_4_) C, H, N.

Further elution of the above column with 4:1 EtOAc/petroleum ether gave impurities, and then elution with EtOAc gave crude oxazine **78** (0.46 g), which was chromatographed again on silica gel. Elution with 0–0.4% MeOH/CH_2_Cl_2_ first gave foreruns, and then elution with 0.5% MeOH/CH_2_Cl_2_ gave purified **78** (305 mg, 11%) as a cream solid (see data below).

*(7R)-7-{[(6-Bromopyridin-3-yl)oxy]methyl}-2-nitro-6,7-dihydro-5H-imidazo[2,1-b][1,3]oxazine (**78**)*. Reaction of alcohol **77** with NaH (1.4 equiv), using procedure D (but extracting the product four times with 10% MeOH/CH_2_Cl_2_ and then four times with CH_2_Cl_2_), followed by chromatography of the product on silica gel, eluting with 0–0.5% MeOH/CH_2_Cl_2_ (foreruns) and then with additional 0.5% MeOH/CH_2_Cl_2_, gave **78** (94%) as a cream solid: mp (MeOH/CH_2_Cl_2_/hexane) 211–212 °C. ^1^H NMR [(CD_3_)_2_SO] *δ* 8.19 (d, *J* = 3.1 Hz, 1 H), 8.10 (s, 1 H), 7.59 (br d, *J* = 8.7 Hz, 1 H), 7.47 (dd, *J* = 8.8, 3.2 Hz, 1 H), 4.96–4.89 (m, 1 H), 4.43 (dd, *J* = 11.2, 3.2 Hz, 1 H), 4.37 (dd, *J* = 11.2, 5.8 Hz, 1 H), 4.18 (ddd, *J* = 12.5, 5.8, 2.9 Hz, 1 H), 4.09 (ddd, *J* = 12.5, 10.9, 5.2 Hz, 1 H), 2.35–2.26 (m, 1 H), 2.25–2.13 (m, 1 H). [*α*]^24^_D_ –61.9 (*c* 1.002, DMF). Anal. (C_12_H_11_BrN_4_O_4_) C, H, N.

*(7R)-7-({[6-(4-Fluorophenyl)pyridin-3-yl]oxy}methyl)-2-nitro-6,7-dihydro-5H-imidazo[2,1-b][1,3]oxazine (**79**)*. Reaction of bromide **78** with 4-fluorophenylboronic acid (1.9 equiv) and Pd(dppf)Cl_2_ (0.25 equiv), using procedure N at 87 °C for 200 min (but extracting the product three times with 10% MeOH/CH_2_Cl_2_ and then three times with CH_2_Cl_2_), followed by chromatography of the product on silica gel, eluting with 0–0.5% MeOH/CH_2_Cl_2_ (foreruns) and then with 0.5–0.67% MeOH/CH_2_Cl_2_, gave **79** (87%) as a cream solid: mp (MeOH/CH_2_Cl_2_/hexane) 205–208 °C. ^1^H NMR [(CD_3_)_2_SO] *δ* 8.43 (d, *J* = 2.9 Hz, 1 H), 8.11 (s, 1 H), 8.07 (br dd, *J* = 8.9, 5.5 Hz, 2 H), 7.94 (d, *J* = 8.8 Hz, 1 H), 7.56 (dd, *J* = 8.8, 3.0 Hz, 1 H), 7.28 (br t, *J* = 8.9 Hz, 2 H), 5.00–4.91 (m, 1 H), 4.47 (dd, *J* = 11.2, 3.2 Hz, 1 H), 4.41 (dd, *J* = 11.2, 5.8 Hz, 1 H), 4.20 (ddd, *J* = 12.5, 5.7, 2.9 Hz, 1 H), 4.11 (ddd, *J* = 12.4, 11.0, 5.2 Hz, 1 H), 2.39–2.29 (m, 1 H), 2.29–2.16 (m, 1 H). [*α*]^23^_D_ –62.6 (*c* 1.006, DMF). Anal. (C_18_H_15_FN_4_O_4_) C, H, N.

**Compounds of Table 2**. The following section details the syntheses of compounds **142** and **160** of Table 2, via representative procedures and key intermediates, as described in Scheme 5. For the syntheses of all of the other compounds in Table 2, please refer to the Supporting Information.

*Synthesis of **142** (Scheme 5A). Procedure S: 4-(2-Chloro-4-nitro-1H-imidazol-1-yl)-1-[4-(4-fluorophenyl)piperazin-1-yl]butan-2-ol (**141**)*. A mixture of epoxide **67** (see the Supporting Information) (150 mg, 0.689 mmol) and 1-(4-fluorophenyl)piperazine (**140**) (186 mg, 1.03 mmol) in MEK (3 mL) in a sealed vial was stirred at 70 °Cfor51 h. The resulting cooled mixture was transferred to a flask (in CH_2_Cl_2_) and evaporated to dryness under reduced pressure (at 30 °C), and then the residue was chromatographed on silica gel. Elution with 0–0.3% MeOH/CH_2_Cl_2_ first gave foreruns, and then further elution with 1–2% MeOH/CH_2_Cl_2_ gave **141** (225 mg, 82%) as a pale-yellow oil. ^1^H NMR (CDCl_3_) *δ* 7.87 (s, 1 H), 6.97 (br dd, *J* = 9.2, 8.3 Hz, 2 H), 6.87 (br dd, *J* = 9.2, 4.6 Hz, 2 H), 4.27 (dd, *J* = 8.2, 5.7 Hz, 2 H), 3.67–3.58 (m, 1 H), 3.57 (v br s, 1 H), 3.19–3.07 (m, 4 H), 2.85–2.77 (m, 2 H), 2.59–2.51 (m, 2 H), 2.41 (dd, *J* = 12.3, 4.0 Hz, 1 H), 2.37 (dd, *J* = 12.3, 9.5 Hz, 1 H), 1.98–1.87 (m, 1 H), 1.82–1.70 (m, 1 H). HRESIMS calcd for C_17_H_22_ClFN_5_O_3_ *m*/*z* [M + H]^+^ 400.1366, 398.1390, found 400.1370, 398.1397.

*7-{[4-(4-Fluorophenyl)piperazin-1-yl]methyl}-2-nitro-6,7-dihydro-5H-imidazo[2,1-b][1,3]oxazine (**142**)*. Reaction of alcohol **141** with NaH, using procedure D at 40 °C for 2 h, followed by chromatography of the product on silica gel, eluting with 0–0.5% MeOH/CH_2_Cl_2_ (foreruns) and then with 1% MeOH/CH_2_Cl_2_, gave **142** (67%) as a pale-yellow solid: mp (CH_2_Cl_2_/hexane) 213–215 °C. ^1^H NMR [(CD_3_)_2_SO] *δ* 8.07 (s, 1 H), 7.04 (br dd, *J* = 9.2, 8.6 Hz, 2 H), 6.94 (br dd, *J* = 9.3, 4.7 Hz, 2 H), 4.79–4.68 (m, 1 H), 4.13 (ddd, *J* = 12.6, 5.9, 2.9 Hz, 1 H), 4.05 (ddd, *J* = 12.5, 10.8, 5.1 Hz, 1 H), 3.13–3.03 (m, 4 H), 2.73 (dd, *J* = 13.5, 6.6 Hz, 1 H), 2.70–2.60 (m, 5 H), 2.29–2.19 (m, 1 H), 2.09–1.95 (m, 1 H). ^13^C NMR [(CD_3_)_2_SO] *δ* 156.0 (d, *J_C–F_* = 235.6 Hz), 148.0, 147.9 (d, *J_C–F_* = 1.6 Hz), 142.1, 117.7, 117.1 (d, *J_C–F_* = 7.6 Hz, 2 C), 115.2 (d, *J_C–F_* = 21.7 Hz, 2 C), 76.0, 60.5, 53.2 (2 C), 49.0 (2 C), 41.8, 24.3. Anal. (C_17_H_20_FN_5_O_3_)C,H,N.

*Synthesis of **160** (Scheme 5C). Procedure T: (2-Nitro-6,7-dihydro-5H-imidazo[2,1-b][1,3]oxazin-7-yl)methyl 4-(4-fluorophenyl)-piperazine-1-carboxylate (**160**)*. Triphosgene (145 mg, 0.489 mmol) was added to a mixture of alcohol **13** (192 mg, 0.964 mmol) and triethylamine (0.40 mL, 2.87 mmol) in anhydrous THF (15 mL). The mixture was stirred at 20 °C for 30 min, and then a solution of 1-(4-fluorophenyl)piperazine (**140**) (347 mg, 1.93 mmol) in anhydrous THF (5 mL) was added. The resulting mixture was stirred at 20 °Cfor 2 h and then quenched with saturated NH_4_Cl (100 mL) and extracted with EtOAc (2 × 100 mL). The combined extracts were evaporated to dryness under reduced pressure, and the residue was chromatographed on silica gel. Elution with 0–2% MeOH/CH_2_Cl_2_ gave crude material, which was successively recrystallized from CH_2_Cl_2_/hexane and EtOAc/hexane, to give **160** (130 mg, 33%) as a cream solid: mp 177–180 °C. ^1^H NMR [(CD_3_)_2_SO] *δ* 8.08 (s, 1 H), 7.06 (br dd, *J* = 9.1, 8.7 Hz, 2 H), 6.97 (br dd, *J* = 9.2, 4.7 Hz, 2 H), 4.84–4.73 (m, 1 H), 4.37 (dd, *J* = 12.2, 3.2 Hz, 1 H), 4.29 (dd, *J* = 12.2, 5.8 Hz, 1 H), 4.15 (ddd, *J* = 12.5, 5.8, 2.7 Hz, 1 H), 4.06 (ddd, *J* = 12.4, 11.3, 5.1 Hz, 1 H), 3.61–3.45 (m, 4 H), 3.14–2.98 (m, 4 H), 2.30–2.20 (m, 1 H), 2.17–2.03 (m, 1 H). ^13^C NMR [(CD_3_)_2_SO] *δ* 156.3 (d, *J_C–F_* = 236.3 Hz), 154.1, 147.8, 147.7 (d, *J_C–F_* = 1.6 Hz), 142.0, 117.9 (d, *J_C–F_* =7.5 Hz, 2 C), 117.7, 115.3 (d, *J_C–F_* = 22.0 Hz, 2 C), 75.8, 65.5, 49.1 (2 C), 43.4 (2 C), 41.7, 22.3. Anal. (C_18_H_20_FN_5_O_5_) C, H, N.

**Minimum Inhibitory Concentration Assays (MABA and LORA)**. These were carried out according to the published protocols.^41,56^

**In Vitro Parasite Growth Inhibition Assays**. The activity of test compounds against the amastigote stage of the *L. don* parasite was assessed at CDRI using a mouse macrophage-based luciferase assay, performed according to the reported procedures.^10^ Further assays measuring the growth inhibitory action of compounds against *L. inf, T. cruzi*, and *T. brucei*, and determining any cytotoxic effects on human lung fibroblasts (MRC-5 cells), were conducted at the University of Antwerp (LMPH), as detailed in a recent article.^40^

**Solubility Determinations**. *Method A*. The solid compound sample was mixed with water or 0.1 M HCl (enough to make a 2 mM solution) in an Eppendorf tube, and the suspension was sonicated for 15 min and then centrifuged at 13000 rpm for 6 min. An aliquot of the clear supernatant was diluted 2-fold with water (or 0.1 M HCl), and then HPLC was conducted. The kinetic solubility was calculated by comparing the peak area obtained with that from a standard solution of the compound in DMSO (after allowing for varying dilution factors and injection volumes).

*Method B*. The thermodynamic solubility of compound **4** at pH 7.4 was measured by Drugabilis, 5 rue Jean-Baptiste Clément, 92290 Châtenay-Malabry, France. The dry powder was stirred with 0.12 M phosphate buffer (pH 7.4) at 20 °C for 24 h. After filtration using a 0.22 *μ*m PVDF membrane filter, the concentration of **4** was determined by HPLC with reference to a standard solution; the final value is the mean from two independent assays.

*Method C*. The thermodynamic solubility of compound **79** at pH 6.5 and 5.0 was measured by WuXi AppTec (Shanghai) Co., Ltd., 288 FuTe ZhongLu, WaiGaoQiao Free Trade Zone, Shanghai 200131, China. Aliquots of the compound DMSO stock (10 mM) were transferred to fasted state simulated intestinal fluid buffer (pH 6.5) or fed state simulated intestinal fluid buffer (pH 5.0), and the mixtures were shaken for 24 h at room temperature. Following sampling by a Whatman filter device, the compound concentrations were determined by UV spectroscopy with reference to three calibration standards (2, 100, and 200 *μ*M).

*Method D*. The thermodynamic solubility of compound **79** at pH 7.4 was measured by Syngene International Ltd., Plot No. 2 and 3 Biocon Park, Jigani Link Rd, Bangalore 560099, India. The dry powder was equilibrated with 0.1 M phosphate buffer (pH 7.4) in a glass vial at 25 °C (water bath), shaking for 24 h. After filtration using a 0.45 *μ*m PVDF membrane filter, the concentration of **79** was determined by HPLC, comparing the peak area obtained with that from a standard solution (0.86 *μ*M) in 1:1:2 EtOH/water/CH_3_CN.

**Microsomal Stability Assays**. Tests on initial compounds **14**, **29**, **34**, **38**, and **39** (Table 3) were run by MDS Pharma Services, 22011 30th Drive SE, Bothell, WA 98021-4444, as previously described.^58^ Compounds **39**, **44**, **49**, **53**, **54**, **59**, **61**, **71**, **107**, **108**, **111–113**, and **116** were evaluated by Advinus Therapeutics Ltd., 21 and 22 Phase II, Peenya Industrial Area, Bangalore 560058, India, using a published procedure^54^ in which the compound concentration was 0.5 *μ*M and the incubation time was 30 min. Additional analyses on compounds **28**, **44**, **59**, **62**, **71**, **79**, **87**, **90**, **91**, **93–95**, **129**, **142**, **152**, **170**, and **178** were performed by WuXi AppTec (Shanghai) Co., Ltd., 288 FuTe ZhongLu, WaiGaoQiao Free Trade Zone, Shanghai 200131, China, via a reported method.^11^

**Distribution Coefficient and p*K*_a_ Measurements**. The octanol–water partition coefficient (Log *P*) of **4** at 20 °C was measured in duplicate by Advinus Therapeutics Ltd., Bangalore, India, using the shake-flask method with HPLC analysis. Log *D* and p*K_a_* data for **79** were measured by WuXi AppTec (Shanghai) Co., Ltd. The Log *D* value was found by assessing the distribution of **79** between 100 mM phosphate buffer of pH 7.4 and octanol at room temperature (final matrix contained 1% DMSO), using the shake-flask method and LC-MS/MS analysis. The p*K_a_* value was obtained by UV spectroscopy, employing 80% MeOH as the initial cosolvent.

**Plasma Protein Binding Assay**. Studies of **4** and **79** were conducted by WuXi AppTec (Shanghai) Co., Ltd., using equilibrium dialysis across a semipermeable membrane. Briefly, a 2 *μ*M compound solution in plasma (0.5% DMSO) was dialyzed against 100 mM phosphate buffered saline (pH 7.4) on a rotating plate incubated for 4 or 6 h at 37 °C. Following precipitation of protein with CH_3_CN, the amount of compound present in each compartment was quantified by LC-MS/MS; values are the mean of triplicate determinations.

**Permeability Assay**. This was performed by WuXi AppTec (Shanghai) Co., Ltd. MDCK-MDR1 cells were seeded onto polyethylene membranes in 96-well plates at 2 × 10^5^ cells/cm^2^, giving confluent cell monolayer formation over 4–7 d. A solution of **79** (2 *μ*M in 0.4% DMSO/HBSS buffer) was applied to the apical or basolateral side of the cell monolayer. Permeation of the compound from A to B direction or B to A direction was determined in triplicate over a 150 min incubation at 37 °C and 5% CO_2_ (95% humidity). In addition, the efflux ratio of **79** was also determined. Test and reference compounds were quantified by LC-MS/MS analysis based on the peak area ratio of analyte/internal standard.

**Ames Test**. Compound **71** (at doses of 1.5, 4, 10, 25, 64, 160, 400, and 1000 *μ*g/well) was evaluated in the Mini-Ames reverse mutation screen conducted by WuXi AppTec (Suzhou) Co., Ltd., 1318 Wuzhong Avenue, Wuzhong District, Suzhou 215104, China. Two *Salmonella* strains (TA98 and TA100) were employed, both in the presence and absence of metabolic activation (rat liver S9). Positive controls (2-aminoanthracene, 2-nitrofluorene, and sodium azide) and a negative (DMSO solvent) control were included.

**hERG Assay**. The effects of compounds **44** and **79** on cloned hERG potassium channels expressed in Chinese hamster ovary cells were assessed by WuXi AppTec (Shanghai) Co., Ltd., using the automated patch clamp method. Six concentrations (0.12, 0.37, 1.11, 3.33, 10, and 30 *μ*M) were tested (at room temperature), and at least three replicates were obtained for each.

**CYP3A4 Inhibition Assay**. The study was performed by WuXi AppTec (Shanghai) Co., Ltd. Compound **79** (at concentrations of 1 and 10 *μ*M) was incubated with NADPH-fortified pooled HLM (0.2 mg/mL) and testosterone (50 *μ*M) in phosphate buffer (100 mM) at 37 °C for 10 min. Following quenching with CH_3_CN, samples were analyzed for the formation of 6*β*-hydroxytestosterone by LC-MS/MS and the percentage inhibition was determined (ketoconazole was the positive control and tolbutamide was used as an internal standard).

**In Vivo Experiments**. All animal experiments were performed according to institutional ethical guidelines for animal care. Antitubercular efficacy studies in mice were approved by the UIC IACUC (UIC AWA no. A3460-01; ACC application no. 12-183). For VL, mouse model studies (LSHTM) were conducted under license from the UK Home Office (license no. PIL 70/6997), hamster studies at CDRI were approved by the CSIR-CDRI animal ethics committee (license no. 19/2009/PARA/IAEC), and hamster studies at LMPH were approved by the ethical committee of the University of Antwerp (UA-ECD 2010-17).

**Acute TB Infection Assay**. Each compound (including **6**, which was employed as an internal reference standard) was administered orally to a group of 7 *M. tb*-infected BALB/c mice at 100 mg/kg daily for 5 days a week for three weeks, beginning on day 11 postinfection, in accordance with published protocols.^41,58^ The results were typically recorded as the ratio of the average reduction in colony forming units (CFUs) in the compound-treated mice/the average CFU reduction in the mice treated with **6**.

**Acute VL Infection Assay (Mouse Model, LSHTM)**. Test compounds were orally dosed once per day for 5 days consecutively to groups of five female BALB/c mice infected with 2 × 10^7^ *L. donovani* amastigotes, with treatment commencing 1 week postinfection, as described.^10^ Miltefosine (**1**) and AmBisome were positive controls, and parasite burdens were determined from impression smears of liver sections. Efficacy was expressed as the mean percentage reduction in parasite load for treated mice in comparison to untreated (vehicle-only) controls.

**Chronic VL Infection Assay 1 (Hamster Model, CDRI)**. Golden hamsters (weighing 40–45 g) were infected intracardially with 1 × 10^7^ *L. donovani* amastigotes, and then, 15 days later, all animals were subjected to splenic biopsy to assess the level of infection. Groups of hamsters having an appropriate infection grading (5–15 amastigotes/100 spleen cell nuclei) were treated with test compounds, starting on day 17 and dosing orally once per day for 5 days, according to the usual procedure.^10^ Post-treatment splenic biopsies taken 12 days after the first dose were employed to determine the intensity of infection, as previously reported.^10^

**Chronic VL Infection Assay 2 (Hamster Model, LMPH)**. Golden hamsters (weighing 75–80 g) were infected with 2 × 10^7^ *L. infantum* amastigotes, and 21 days postinfection, treatment groups of 6 animals each were dosed orally once or twice per day with test compounds (formulated in PEG-400) for 5 days consecutively. Parasite burdens in three target organs (liver, spleen, and bone marrow) were determined by microscopic evaluation of impression smears (stained with Giemsa), and efficacy was expressed as the mean percentage load reduction for treated hamsters in comparison to untreated (vehicle-only) controls. Miltefosine (**1**) was included as a reference drug in all experiments.

**Mouse Pharmacokinetics**. Compound **28** was evaluated by UNT Health Science Center, 3500 Camp Bowie Boulevard, Fort Worth, TX 76107-2699 (using a method approved by the UNTHSC IACUC; AWA no. A3711–01). Following oral administration to female BALB/c mice at 40 mg/kg as a suspension in 0.5% carboxymethylcellulose/water, blood samples were collected (at time intervals of 0.5, 1, 1.5, 2, 4, 6, 8, and 24 h), centrifuged, and analyzed by LC-MS/MS to generate the required PK parameters. Compound **34** was assessed by MDS Pharma Services, 22002 26th Avenue SE, Suite 104, Bothell, WA 98021-4444, via a similar procedure (but employing mixed gender CD-1 mice and an oral formulation of 0.5% carboxymethylcellulose and 0.08% Tween 80 in water). Studies of compounds **39**, **44**, **49**, **53**, **54**, and **112** were conducted by Advinus Therapeutics Ltd., 21 and 22 Phase II, Peenya Industrial Area, Bangalore 560058, India, according to a published protocol.^54^ Briefly, compounds were administered to groups of male Swiss Albino mice; intravenous dosing (at 1 mg/kg) employed a solution vehicle comprising 20% NMP and 40% PEG-400 in 100 mM citrate buffer, pH 3, while oral dosing (at 25 mg/kg) was as a suspension in 0.5% carboxymethylcellulose and 0.08% Tween 80 in water (except for **112**, where the iv solution was 10% NMP, 10% cremophor EL and 10% propylene glycol in saline, and oral dosing at 12.5 mg/kg was as a suspension in 7% Tween 80 and 3% EtOH in water). Samples derived from plasma (at 0.083 for iv only, 0.25, 0.5, 1, 2, 4, 6, 8, 10, 24, and 48 h) were centrifuged prior to analysis by LC-MS/MS, and the PK parameters were determined using WinNonlin software (version 5.2). Finally, **71** was examined by WuXi AppTec (Shanghai) Co., Ltd.; in this case, oral dosing of female BALB/c mice was at 25 mg/kg in PEG-400 (sampling at 0.25, 1, 2, 4, 8, and 24 h), and the PK data were derived following similar LC-MS/MS analysis.

**Rat and Hamster Pharmacokinetics**. Compounds **71**, **79**, and **87** were assessed in male Sprague–Dawley rats and female golden Syrian hamsters by WuXi AppTec (Shanghai) Co., Ltd. Intravenous dosing (at 1 mg/kg for rats and 2 mg/kg for hamsters) utilized a solution formulation of 20% NMP and 40% PEG-400 in citrate buffer, pH 3. In rats, oral dosing (at 5 mg/kg) was as a suspension in 0.08% Tween 80 and 0.5% carboxymethylcellulose in water, whereas PEG-400 was the vehicle employed for oral dosing in hamsters (at 12.5 mg/kg). Plasma samples (at 0.083 for iv only, 0.25, 0.5, 1, 2, 4, 8, and 24 h) were analyzed by LC-MS/MS, and the PK parameters were calculated using WinNonlin software (version 6.3).

## ■ ASSOCIATED CONTENT

### Supporting Information

The Supporting Information is available free of charge on the ACS Publications website at DOI: 10.1021/acs.jmedchem.7b00034.

Additional biological assay data, synthetic schemes, graphs of PK and assay data, experimental procedures and characterizations for compounds, combustion analytical data, and representative NMR spectra (PDF) Molecular formula strings spreadsheet (CSV)

## ■ AUTHOR INFORMATION

### ORCID

Andrew M. Thompson: 0000-0003-2593-8559

### Notes

The authors declare no competing financial interest.

## ■ ABBREVIATIONS USED

VL,: visceral leishmaniasis;
TB,: tuberculosis;
MEK,: methyl ethyl ketone (2-butanone);
*L. don*,: *Leishmania donovani*;
*L. inf*,: *Leishmania infantum*;
*T. cruzi*,: *Trypanosoma cruzi*;
*T. brucei*,: *Trypanosoma brucei*;
*rac*,: racemic;
MLM,: mouse liver microsomes;
PK,: pharmacokinetic;
PPB,: plasma protein binding;
PD,: pharmacodynamic;
*M. tb*,: *Mycobacterium tuberculosis*;
HRFABMS,: high resolution fast atom bombardment mass spectrometry;
HRESIMS,: high resolution electrospray ionization mass spectrometry;
APCI MS,: atmospheric pressure chemical ionization mass spectrometry;
HLM,: human liver microsomes;
CFU,: colony forming unit;
SD,: standard deviation
